# Micropatterned Composite Hydrogel Sheet with Surface Electronic Conductive Network for Ultrasensitive Strain Sensing

**DOI:** 10.3390/gels11110913

**Published:** 2025-11-15

**Authors:** Ruidong Chu, Mingyu Liu, Wenxia Liu, Zhaoping Song, Guodong Li, Dehai Yu, Xiaona Liu, Huili Wang

**Affiliations:** State Key Laboratory of Green Papermaking and Resource Recycling, Qilu University of Technology, Shandong Academy of Science, Jinan 250353, China

**Keywords:** composite hydrogel sheet, micropattern, stress concentration, carbon nanotubes, strain sensing

## Abstract

Conductive hydrogels show great promise for wearable sensors but suffer from low sensitivity in small strain ranges. In this study, we developed a micropatterned composite hydrogel sheet (thickness: 1.2 ± 0.1 mm) by constructing a continuous electronic conductive network of carbon nanotubes (CNTs) on a highly crosslinked micropatterned hydrogel sheet. The sheet was fabricated via a two-step synthesis of a polyvinyl alcohol/polyacrylic acid polymer network—crosslinked by Zr^4+^ in a glycerol-water system—using sandpaper as the template. The first step ensured tight conformity to the template, while the second step preserved the micropattern’s integrity and precision. The reverse sandpaper micropattern enables secure bonding of CNTs to the hydrogel and induces localized stress concentration during stretching. This triggers controllable cracking in the conductive network, allowing the sensor to maintain high sensitivity even in small strain ranges. Consequently, the sensor exhibits ultra-high sensitivity, with gauge factors of 76.1 (0–30% strain) and 203.5 (30–100% strain), alongside a comfortable user experience. It can detect diverse activities, from subtle physiological signals and joint bending to complex hand gestures and athletic postures. Additionally, the micropatterned composite hydrogel sheet also demonstrates self-healing ability, adhesiveness, and conformability, while performing effectively under extreme temperatures and sweaty conditions. This innovative structure and sensing mechanism—leveraging stress concentration and controlled crack formation—provides a strategy for designing wearable electronics with enhanced performance.

## 1. Introduction

Conductive hydrogels—composed of cross-linked polymer networks and conductive materials swollen in water—are soft conductors with inherent stretchability, tunable mechanical and electronic properties, and capabilities for self-healing and adhesion [1,2,3]. These attributes allow them to conform tightly to human skin, maintaining stable conductivity while resisting damage. Consequently, they are ideal for flexible strain sensors, finding applications in human motion detection [4,5], electronic skin [6,7,8,9,10,11], medical rehabilitation [12,13], and intelligent robotics [14]. Notably, hydrogels modified with organic solvents, anti-freezing agents, or inorganic salts [15,16,17,18,19,20] can operate effectively under extreme temperatures. This versatility has driven intense interest in conductive hydrogel-based strain sensors, fueling their rapid development in the wearable technology [21,22].

Conductive materials—such as ionic electrolytes and conductive fillers—are commonly incorporated into hydrogels to impart conductivity, a key factor in enhancing the sensing performance of hydrogel-based strain sensors, particularly their sensitivity. However, simply mixing these materials with hydrogel precursors often limits their incorporation within the polymeric network, resulting in low conductivity and relatively poor sensitivity, especially under small strains [23,24]. To address this, cellulose fiber-derived carbon aerogels have been directly embedded into conductive hydrogels, forming continuous electronic conductive networks that significantly boost sensitivity for strain sensing [25]. Replacing these aerogels with those made of highly conductive materials—such as carbon nanotubes (CNTs)—further boosts the composite hydrogel’s conductivity and, in turn, its sensitivity [26]. Additionally, integrating carbonized crepe paper (CCP)—with its corrugations aligned perpendicular to the stretching direction—into hydrogels creates a composite with a corrugated conductive network, enabling substantial sensitivity improvements even at low strain ranges [27].

However, fabricating CCP-based composite hydrogels with ultra-high sensitivity requires precise alignment between CCP and the encapsulating hydrogel to trigger crack formation at low strains [27,28,29]. Additionally, hydrogel penetration into the continuous conductive networks can drastically reduce the composite’s conductivity, limiting further sensitivity improvements for strain sensing applications. To address this, depositing a conductive carbon nanotube (CNT) film or coating a layer of reduced graphene oxide onto the hydrogel surface maximizes conductivity, enabling composite hydrogel-based strain sensors to achieve substantially higher sensitivity [30,31]. Despite these advances, the stability and durability of such sensors remain problematic due to the exfoliation of conductive materials [32,33].

Hydrogels with surface micropatterns have been widely used to develop flexible pressure sensors, as they effectively expand deformation ranges [34,35,36]. However, their application in strain sensor fabrication remains limited. In this study, we fabricated a micropatterned hydrogel sheet using sandpaper as a template [37,38]—a design that differs from pressure sensor applications: here, the micropattern primarily serves to anchor CNTs and induce controllable stress concentration during stretching. This mechanism enables the formation of a dense, continuous conductive network on the hydrogel surface, amplifies local deformation, and enhances sensing performance. To replicate the sandpaper micropattern and create a tough, self-healing, self-adhesive hydrogel sheet with excellent temperature tolerance, we constructed a dual-polymer network: polyvinyl alcohol (PVA) combined with in situ synthesized polyacrylic acid (PAA), cross-linked by Zr^4+^ ions in a glycerol-water solvent via a two-step gelatinization method. Coating the micropatterned surface of this PVA-PAA-Zr^4+^ hydrogel with a CNT layer yielded a composite hydrogel sheet featuring an interwoven, tightly bonded conductive network. The reverse sandpaper micropattern significantly increases the surface area, exposing more reactive sites for CNT interaction and generating mechanical interlocking—greatly enhancing CNT adhesion to the hydrogel surface. The inherent rigidity of CNTs, coupled with their strong adhesion to the hydrogel, improves the composite’s robustness, durability, and stability. Notably, the micropatterned composite hydrogel sheet exhibits ultra-high sensitivity even at low strains: this is attributed to the high conductivity of the tight conductive network, as well as the controllable microcrack formation at the edges of irregular protrusions and depressions (driven by stress concentration during stretching). With its excellent processability, the micropatterned composite hydrogel sheet is an ideal candidate for health monitoring and human detection applications.

## 2. Results and Discussion

### 2.1. Preparation and Characterization

**Figure 1:**
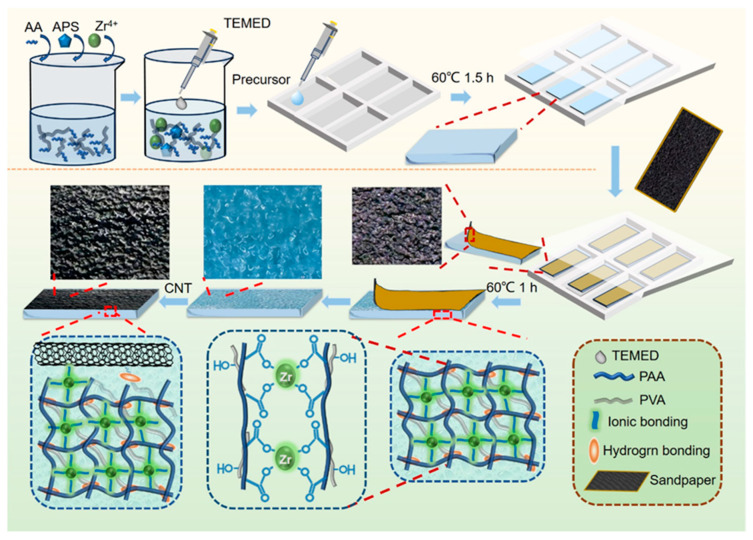
Specific preparation process of the micropatterned composite hydrogel sheet.

As shown in Figure 1, the micropatterned composite hydrogel sheet was prepared by coating a multi-walled carbon nanotube (MWCNT) aqueous dispersion onto a micropatterned PVA-PAA-Zr^4+^ hydrogel sheet. The latter was fabricated via a two-step polymerization/gelatinization process using hydrogel precursor filled in a polytetrafluoroethylene (PTFE) mold, with details as follows:

In the first step of gelatinization, most acrylic acid (AA) dissolved in a glycerol-water binary solvent system was converted to polyacrylic acid (PAA) in the presence of PVA and ZrCl_4_. This free radical polymerization was initiated by ammonium persulfate (APS), catalyzed by N,N,N′,N′-tetramethylethylenediamine (TEMED), and conducted at 60 °C for 1.5 h. The in situ formed PAA was crosslinked simultaneously by Zr^4+^—via coordination with PAA’s carboxyl groups—and by hydrogen bonding between PAA’s carboxyl groups and PVA’s hydroxyl groups. Owing to the shortened reaction time, this yielded a preformed hydrogel sheet with a PVA-PAA dual polymer network structure. A smooth glass sheet was placed on the PTFE mold as a template to ensure the preformed sheet had a flat surface. The resulting first-step product exhibited a smooth surface and soft texture. After removing the glass slide, sandpaper (Figure S1a) matching the mold dimensions was pressed onto the preformed hydrogel’s smooth surface. The second gelatinization step was then performed at 60 °C for 1 h, enhancing the hydrogel’s modulus to precisely retain the micropattern without significant distortion. Following sandpaper removal, a PVA-PAA-Zr^4+^ hydrogel sheet (thickness: ~1.0–1.1 mm) with an inverse sandpaper micropattern on its surface, was obtained (Figure S1b).

Subsequently, an MWCNT aqueous dispersion was coated onto the micropatterned surface of the PVA-PAA-Zr^4+^ hydrogel sheet. Water in the dispersion induced slight swelling of the hydrogel, which enhanced surface wettability towards MWCNTs and created a favorable environment for the interactions between MWCNTs and the hydrogel’s surface components (PAA, PVA, and Zr^4+^). As water naturally evaporated from the dispersion, MWCNTs gradually concentrated on the hydrogel sheet surface. This concentration effect promoted stronger intermolecular interactions: not only between MWCNTs (via their oxygen-containing functional groups) but also between MWCNTs and the hydrogel’s PAA, PVA, and Zr^4+^. These interactions were mediated primarily by hydrogen bonding and Zr^4+^ coordination (Figure 1).

**Figure 2:**
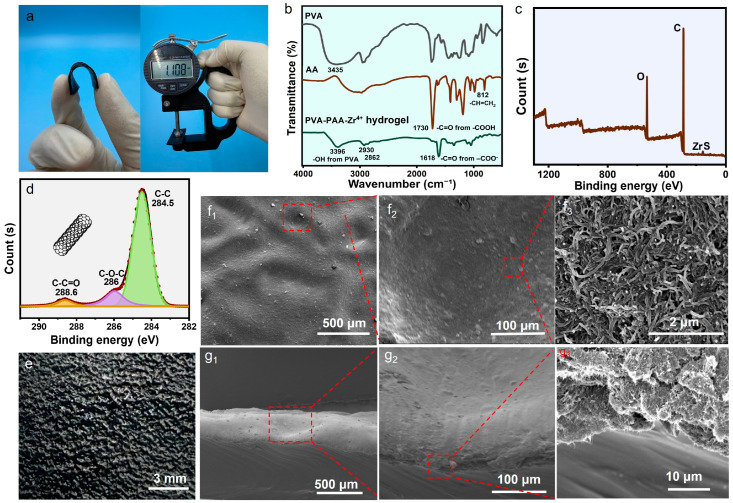
(**a**) Photograph illustrating the flexibility and thickness of the micropatterned composite hydrogel sheet. (**b**) Infrared spectra of PVA, AA and the PVA-PAA-Zr^4+^ hydrogel sheet. (**c**) XPS survey spectrum of the PVA-PAA-Zr^4+^ hydrogel sheet. (**d**) C 1s XPS spectrum of MWNCTs. (**e**) Photograph showing the surface micropattern of the micropatterned composite hydrogel sheet. (**f_1_**–**f_3_**) Surface SEM images of the micropatterned composite hydrogel sheet. (**g_1_**–**g_3_**) Cross-sectional SEM images of the micropatterned composite hydrogel sheet.

Furthermore, water evaporation induced shrinkage of the swollen hydrogel sheet. This shrinkage embedded MWCNTs into the hydrogel’s micropattern, further reinforcing interfacial interactions between MWCNTs and the hydrogel matrix. After complete evaporation, a stable, compact MWCNT layer was formed on the PVA-PAA-Zr^4+^ hydrogel’s micropatterned surface—yielding a micropatterned composite hydrogel sheet with a conformably bonded MWCNT coating (Figure S1c). As shown in Figure 2a, the micropatterned composite hydrogel exhibits good flexibility and a thickness of 1.2 mm ± 0.1 mm.

The conversion of AA during fabrication of the PVA-PAA-Zr^4+^ hydrogel sheet was confirmed by comparing its infrared (IR) spectrum with those of AA and PVA. As shown in Figure 2b, the stretching vibration peaks of =CH_2_ (3070–3090 cm^−1^) and =CH– (3010–3040 cm^−1^) in AA’s –HC=CH_2_ group of AA are masked by its broad –OH stretching vibration peak (2500–3300 cm^−1^). In the PVA-PAA-Zr^4+^ hydrogel’s IR spectrum, the –OH stretching vibration is centered at 3396 cm^−1^ due to the introduction of PVA, which exhibits its –OH peak at 3435 cm^−1^. Notably, the 812 cm^−1^ absorption peak (attributed to AA’s –HC=CH_2_ group) is absent in the hydrogel spectrum, while new saturated C–H stretching vibration peaks emerge at 2930 cm^−1^ and 2862 cm^−1^ (enhanced by PVA’s C–H vibrations). This confirms the polymerization of AA into PAA [26] and the presence of PVA. Additionally, AA’s –C=O peak at 1730 cm^−1^ weakens in the hydrogel, likely due to hydrogen bonding and Zr^4+^ coordination in the PVA-PAA dual network. This peak is further overlapped by the 1735 cm^−1^ signal from residual acetate groups in PVA, despite the low PVA-to-AA mass ratio (0.7:2, Table S1). Even so, oxygen-containing groups in the hydrogel sheet remain predominantly as –C=O (Figure S2a). A new peak at 1618 cm^−1^ (Figure 2c and Figure S2b) indicates conversion of AA’s –COOH group to –COO^−^ via coordination with Zr^4+^.

The MWCNTs on the surfaces of the micropatterned composite hydrogel sheets are characteristic graphitic materials, as confirmed by their X-ray diffraction (XRD) pattern (Figure S2c), which shows a distinct (002) diffraction peak of graphite at 2θ = 25.9°. This sharp peak indicates the high crystallinity of the MWCNTs. Their Raman spectrum (Figure S2d) reveals both a defect-induced D-band and a G-band corresponding to highly ordered crystalline carbon structures. The low intensity ratio of the D-band to the G-band (I_D_/I_G_ = 0.69) further confirms the high degree of graphitization.

The high degree of graphitization provides a critical foundation for constructing a continuous and highly conductive electronic network on the hydrogel surface. Additionally, strong interfacial bonding between MWCNTs and the PVA-PAA-Zr^4+^ hydrogel—mediated by carboxyl groups on both components (Figure 2d)—ensures macroscopically uniform distribution of MWCNTs across the composite hydrogel surface (Figure 2e). This uniformity facilitates the formation of a dense, conformally adherent conductive network (Figure 2(f_1_–f_3_)). The micropatterned composite hydrogel sheet retains the surface topology of the pristine PVA-PAA-Zr^4+^ hydrogel sheet, featuring irregular protrusions and depressions. The MWCNT coating has a thickness of 75–100 µm (Figure 2(g_1_,g_2_)); in the cross-sectional view, MWCNTs are stacked in multiple layers, with microcracks formed between the interwoven MWCNT aggregates (Figure 2(g_3_)). This unique structure enables MWCNTs to form a surface-confined three-dimensional conductive networks. Consequently, the micropatterned composite hydrogel sheet achieves an ultra-high conductivity of 27.8 S/m and exhibits significant resistance changes even at low strains—attributed to the formation of these microcracks [39].

### 2.2. Conventional Properties

**Figure 3:**
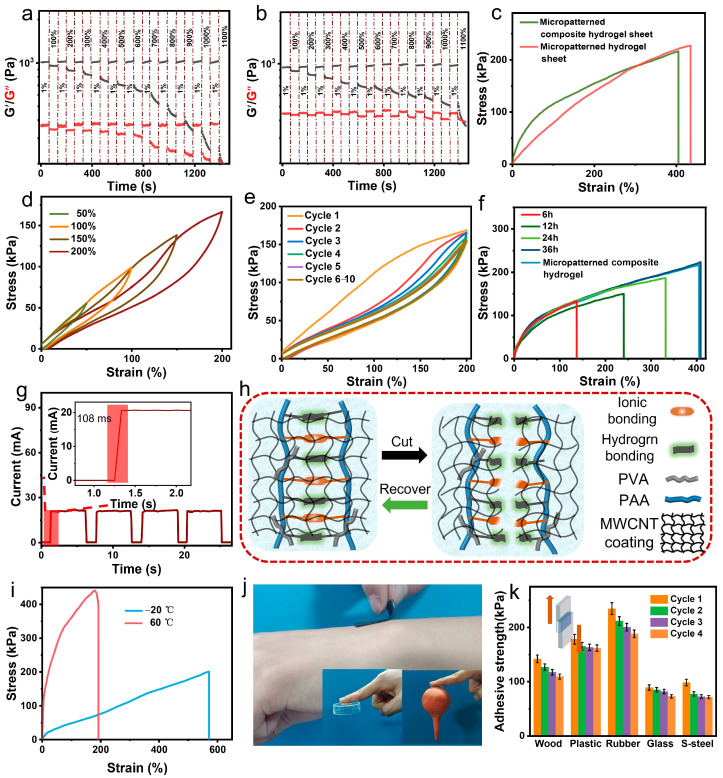
(**a**) Energy storage modulus (G′) and loss modulus (G″) of the micropatterned PVA-PAA-Zr^4+^ hydrogel sheet at alternating step strains (1% and 1100%) measured at a frequency of 6.28 rad/s. (**b**) Energy storage modulus (G′) and loss modulus (G″) of the micropatterned composite hydrogel sheet at alternating step strains (1% and 1100%) measured at a frequency of 6.28 rad/s. (**c**) Stress–strain curves of the micropatterned composite hydrogel sheet and micropatterned hydrogel sheet. (**d**) Cyclic stress–strain curves of the micropatterned composite hydrogel sheet over the strain range from 50% to 200%. (**e**) Cyclic stress–strain curves of the micropatterned composite hydrogel sheet under loading-unloading a 200% strain for 10 cycles. (**f**) Stress–strain curves of the cut micropatterned composite hydrogel sheet (with the fracture surface coated with hydrogel precursor) after self-healing for different durations at room temperature. (**g**) Conductive self-healing time of the micropatterned composite hydrogel sheet. (**h**) Schematic self-healing mechanism of the micropatterned composite hydrogel sheet. (**i**) Stress–strain curves of the micropatterned composite hydrogel sheet conditioned at 60 °C for 2 h and at −20 °C for 24 h. (**j**) Photographs showing the adhesion of the micropatterned composite hydrogel sheet to different substances. (**k**) Adhesion strength of the micropatterned hydrogel sheet to different materials.

As shown in Figure 3a and Figure S3a, both the micropatterned and non-micropatterned PVA-PAA-Zr^4+^ hydrogel sheets exhibit a storage modulus (G′) significantly larger than their loss modulus (G″)—even at an oscillatory strain of up to 900%. This confirms their highly crosslinked structure [40], primarily driven by hydrogen bonding between hydrogel groups in PVA chains and carboxyl groups in PAA chains, as well as Zr^4+^ coordination with PAA’s carboxyl groups. Introducing a surface micropattern via sandpaper templating shifts the crossover point of G′ and G″ from 1000% to 1100% strain. This shift indicates that the surface micropattern induces heterogeneous deformation and localized stress distribution. Through strain hardening, this effect delays the hydrogel’s transition from solid-like to liquid-like behavior by preventing uniform deformation of the entire network.

Furthermore, coating both micropatterned and non-micropatterned hydrogel sheets with MWCNTs had little effect on the oscillatory strain required for the solid-to-sol transition. However, at 1% oscillatory strain, the MWCNT-coated hydrogels exhibited lower G′ but higher G″, with both moduli decreasing more gradually as strain increased (Figure 3b and Figure S3b). This behavior suggests that surface-deposited MWCNTs may slightly disrupt the polymer network at the hydrogel sheet-MWCNTs interface, yet they help preserve the structural integrity of the bulk hydrogel network. This effect stems from the relatively high stiffness of MWCNTs and their strong interfacial bonding with the hydrogel sheet, while the MWCNT network enhances energy dissipation under strain by acting as reversible crosslinking sites.

The highly crosslinked structure of the PVA-PAA-Zr^4+^ hydrogel endows it with a tensile strength of 234.0 kPa and moderate stretchability (elongation at break: 445.8%) (Figure S4a) [41]. Introducing both the surface micropattern and the MWCNT layer stiffens the hydrogel sheet, increasing the Young’s modulus of both the micropatterned pristine hydrogel and the flat MWCNT-coated composite hydrogel (Figure S4b). However, the surface micropattern mitigates the hardening effect of MWCNTs by disrupting the formation of a rigid MWCNT network—enabling the micropatterned composite hydrogel sheet to achieve a moderate Young’s modulus (144.1 kPa) while retaining high stretchability (Figure S5). Notably, the micropatterned composite exhibits an elongation at break exceeding 400% (Figure 3c)—far higher than the maximum strain required for human motion detection. It also maintains excellent toughness: at 0.539 MJ/m^3^, its toughness is nearly comparable to that of the pristine PVA-PAA-Zr^4+^ hydrogel sheet (0.593 MJ/m^3^) (Figure S6).

The cyclic tensile stress–strain curves of the micropatterned composite hydrogel sheet exhibit a hysteresis loop in each cycle, with loading and unloading strains ranging from 50% to 200% (Figure 3d). Larger strains correspond to broader hysteresis loop, confirming that energy dissipation plays a significant role in the composite’s mechanical properties. This energy dissipation originates from the breakage of reversible bonds: hydrogen bonding/ionic coordination within the PVA-PAA-Zr^4+^ hydrogel matrix and hydrogen bonding/physical entanglement in the surface MWCNT layer. As the strain increases from 50% to 200%, the dissipated energy rises from 3.94 kJ/m^2^ to 54.19 kJ/m^2^—attributed to enhanced damage to the crosslinked network.

When the micropatterned composite hydrogel sheet was cyclically loaded and unloaded at 200% strain for 10 cycles (Figure 3e), the unloading curves almost completely overlapped—highlighting the material’s reversible deformation and robust elasticity. The hysteresis loop area in the first cycle is significantly larger than that in subsequent cycles, which can be attributed to the breakage of weaker cross-links, realignment of polymer chains, and untangling of entangled regions [14]. These processes reduce internal energy dissipation in subsequent cycles, resulting in progressively smaller hysteresis loops after the first cycle. By the fifth cycle, the hysteresis loop stabilizes at a relatively small size, demonstrating the composite’s excellent fatigue resistance. Overall, the micropatterned composite hydrogel sheet retains typical hydrogel characteristics, including high resilience and good durability under cyclic mechanical loading.

The micropatterned PVA-PAA-Zr^4+^ composite hydrogel sheet features dynamic crosslinking via reversible ionic coordination and hydrogen bonds, while its MWCNT coating forms via physical interweaving of MWCNTs (mediated by hydrogen bonds and physical contacts). This unique structure endows the material with self-healing capabilities for both mechanical and electronic properties. As shown in Figure S7a, the tensile strength and elongation at break of the cut micropatterned composite hydrogel sheet are progressively restored after butt-jointing at room temperature. However, its high crosslinking degree leads to low mobility of PVA and PAA chains, as well as the hindered migration of water and glycerol to the cut section—resulting in relatively slow mechanical self-healing. To enhance the self-healing rate, a binary glycerol-water solvent can be applied to the cut surface. With this approach, the self-healing rates of tensile strength and elongation at break reach 93.8% and 83.4%, respectively, at room temperature within 36 h (Figure S7b). Heating at 60 °C for 1.5 h further increases these rates to 94.9% and 86.6%, respectively (Figure S7c). Notably, coating the cut surface with hydrogel precursor yields superior results: at room temperature within 36 h, the self-healing rates of tensile strength and elongation at break reach 99.1% and 97.0%, respectively (Figure 3f), approaching near-complete self-healing. Similarly, heating the precursor-coated sample at 60 °C for 1.5 h enables the cut hydrogel’s mechanical properties to approach full recovery (Figure S7d).

Conductivity recovery was characterized by monitoring real-time current changes after repeated cutting and rejoining. As shown in Figure 3g, the composite’s current recovered within 108 ms. This rapid electrical recovery stems from the quick re-establishment of the conductive paths—dominated by physical recontact of surface MWCNTs—after the cut segments are rejoined. MWCNT recontact during healing is primarily driven by van der Waals interactions and capillary forces at the interface. When MWCNTs are brought within ~5 nm, π–π stacking locks them into a stable, reconnected network. This mechanism enables fast conductivity restoration, even after complete severing. In contrast, mechanical property recovery is slower, as it relies on the time required for the broken PVA and PAA chains to reapproach and recrosslink via hydrogen bonding and ionic coordination in the hydrogel’s aqueous environment. When the two cut surfaces are rejoined, van der Waals forces and immediate hydrogen bond formation create weak adhesion to prevent separation. Simultaneously, PVA/PAA chains, and interfacial Zr^4+^ ions diffuse across the boundary, driven by thermal motion and surface energy minimization. As diffusion proceeds, hydrogen bonds form between PVA chains and between PVA and PAA chains, while Zr^4+^ ions re-coordinate with -COO^−^ groups from both segments. This bond re-formation and chain entanglements gradually integrate the two segments into a unified network, reducing interfacial stress concentration and enabling the healed hydrogel to withstand mechanical loads. The final strength depends on the density of re-formed bonds and the extent of chain entanglement, so recovering over 90% of the original tensile strength typically requires a relatively long period. Additionally, the reconnection of surface MWCNTs contributes to this mechanical healing process (Figure 3h).

Glycerol, a typical antifreeze agent, is incorporated into the micropatterned composite hydrogel sheet to enhance temperature tolerance. It forms stronger and more extensive hydrogen bonds with water molecules than the hydrogen bonds between the water molecules themselves. Additionally, glycerol exhibits much lower volatility than water. These characteristics enable exceptional freezing resistance: glycerol disrupts the regular arrangement of water molecules and restricts their aggregation at low temperatures, thereby preventing ice crystal formation. Glycerol also contributes to high-temperature stability. At elevated temperatures, it inhibits water molecule evaporation and reduces water vapor pressure—effectively mitigating the loss of moisture that would otherwise degrade the hydrogel’s structure and performance.

However, water evaporation is inevitable even at room temperature when the micropatterned composite hydrogel sheet is exposed to air (Figure S8), owing to its relatively large surface area. Notably, the hydrogel retained considerable tensile strength and elongation at break after conditioning at 60 °C for 2 h and at −20 °C for 24 h (Figure 3i). At −20 °C, its tensile strength only slightly decreased to 201.1 kPa, while its elongation at break unexpectedly increased to 570.4%. This improvement is attributed to glycerol’s dual roles: its the plasticizing effect and ability to inhibit freezing enhance flexibility, albeit with a slight reduction in stress resistance. In contrast, the hydrogel exhibited more pronounced mechanical property changes in high-temperature environments, driven by water loss. Specifically, its tensile strength significantly increased to 441.7 kPa, while elongation at break decreased to 191.8%.

The micropatterned composite hydrogel sheet, with abundant carboxyl and hydroxyl groups derived from PAA and PVA, exhibits adhesive properties—particularly on its MWCNT-free side. Photographs in Figure 3j and Figure S9 clearly confirm its good adhesion to skin, rubber, glass, and plastic: when applied to the skin, its thinness (1.2 mm) and good permeability (Figure S10) ensure excellent conformability and a comfortable feel [42], while its relatively high Young’s modulus (Figure S4b) allows easy residue-free removal without skin damage (Figure 3j). For quantitative evaluation, lap shear tests were conducted, and as shown in Figure 3k, the hydrogel sheet achieved the strongest adhesion to rubber, followed by plastic, and then wood—attributed to the decreasing surface energy and relatively rough surfaces of these substrates. In contrast, it showed weaker adhesion to glass and stainless steel, which is associated with their higher surface energy and smoother surfaces. After four adhesion-stripping cycles, adhesion to glass decreased the least (benefiting from its smooth surface), while adhesion to rubber exhibited the most significant decay—due to rubber’s rough surface and inherently strong initial adhesion.

Additionally, the micropatterned composite hydrogel sheet exhibits antibacterial properties (Figure S11), which helps prevent biofouling during application. This antibacterial activity is attributed to the presence of Zr^4+^, as evidenced by the following observations: the PVA-PAA hydrogel without Zr^4+^ ions shows no antibacterial effect (Figure S12a,d), while the PVA-PAA-Zr^4+^ hydrogel sheet displays an inhibition zone comparable to that of the micropatterned composite hydrogel sheet (Figure S12b,c,e,f). The antibacterial effect of Zr^4+^ ions in the hydrogel sheet arises from its interference with the metabolic activities of bacterial cells [43].

### 2.3. Sensing Performance

**Figure 4:**
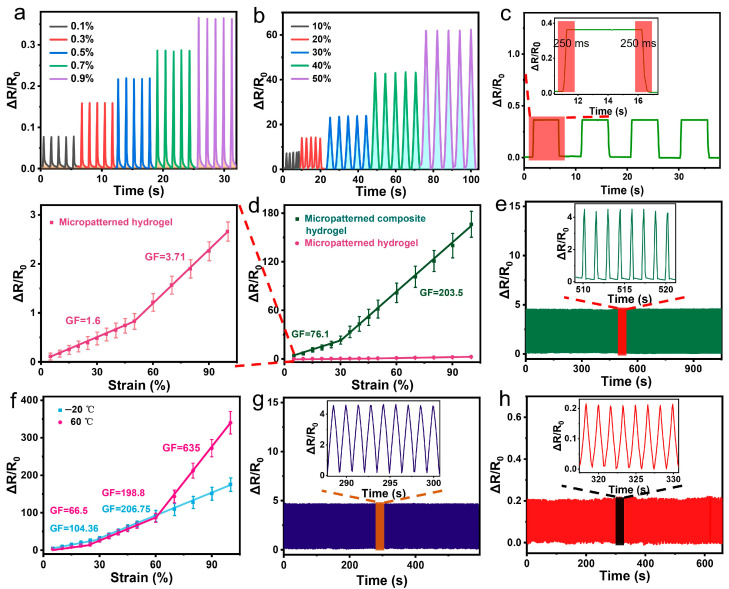
(**a**) Real-time relative resistance change (∆R/R_0_) of the micropatterned composite hydrogel sheet-based strain sensor: in the low strain range (0.1–0.9%). (**b**) Real-time ∆R/R_0_ of the sensor in the moderate strain range (10–50%). (**c**) Real-time ∆R/R_0_ of the sensor at 1% strain, with enlarged waveforms showing the response and recovery times. (**d**) ∆R/R_0_ versus strain curves of strain sensors based on micropatterned PVA-PAA-Zr^4+^ hydrogel sheet and micropatterned composite hydrogel sheet. (**e**) Real-time ∆R/R_0_ of the micropatterned composite hydrogel sheet-based strain sensor under cyclic strain of 5% for 800 cycles at room temperature. (**f**) Real-time ∆R/R_0_ versus strain curves of the strain sensor after conditioning at −20 °C for 24 h and at 60 °C for 2 h. (**g**) Real-time ∆R/R_0_ of the sensor under cyclic strain of 5% for 400 cycles after conditioning at −20 °C for 24 h. (**h**) Real-time ∆R/R_0_ of the sensor under cyclic strain of 5% for 400 cycles after conditioning at 60 °C for 2 h.

The sensing performance of a resistive hydrogel-based strain sensor is generally evaluated by its relative resistance change (∆R/R_0_) under applied strain—this performance can be improved by either enhancing the resistance change (∆R) or reducing the initial resistance (R_0_) of the sensor. The micropatterned composite hydrogel sheet exhibits high conductivity of 27.8 S/m, attributed to the formation of a continuous electronic conductive network enabled by the strong bonding of the surface MWCNT coating. Additionally, the rigidity mismatch between the MWCNT coating and the soft PVA-PAA-Zr^4+^ matrix, combined with the stress concentration induced by the surface micropattern, allows the MWCNT coating to generate microcracks even at low strains [44], which significantly enhances the sensor’s ∆R/R_0_. This combination of high conductivity and substantial ∆R under strain provide an excellent foundation for the composite hydrogel sheet to serve as a highly sensitive strain-sensing material. As shown in Figure 4a,b, the sensor based on this hydrogel outputs regular, repeatable real-time electronic signals, with maximum values proportional to the applied strains both in the small strain range (0.1–0.9%) and the relatively large strain range (10–50%), confirming its excellent performance as a strain sensing material. At 0.1% strain, the relative resistance change (ΔR/R_0_) reaches 7.8%, indicating an extremely low strain detection limit (Table S2). Notably, since 0.1% strain is the smallest strain the texture analyzer can accurately measure, the sensor’s actual strain detection limit is below 0.1%.

From the real-time ΔR/R_0_ data of the sensor based on the micropatterned composite hydrogel sheet, recorded during repeated loading and unloading at 1% strain (Figure 4c), the sensor exhibits a response and recovery time of 250 ms. This indicates well-balanced response and recovery performance (Table S2).

The output signal ∆R/R_0_ of the micropatterned composite hydrogel-based sensor exhibits a two-segment linear relationship with applied strain (0–30% and 30–100%, Figure 4d), corresponding to ultra-high gauge factors (GF) of 76.1 and 203.5, respectively (Table S2). In contrast, both micropatterned and non-micropatterned PVA-PAA-Zr^4+^ hydrogel sheets (without MWCNTs) show GF values below 4.0 across all tested strain ranges (Figure 4d and Figure S12), while the non-micropatterned composite hydrogel (with MWCNTs) displays a relatively low GF of 27.1 at strains <15% but outperforms the micropatterned counterpart in other strain ranges (Figure 4d and Figure S12). These results confirm that the ultra-high sensitivity of the micropatterned composite hydrogel sensor stems from the conductive MWCNT coating—this coating forms a continuous electronic conductive network and induces deformation and cracking during stretching, which together contribute to the remarkable GF performance that distinguishes it from hydrogels without MWCNTs and even non-micropatterned composite counterparts in low-to-moderate strain ranges.

The surface micropattern of the composite hydrogel sheet serves two critical roles: it strengthens the bonding between the MWCNTs and the hydrogel matrix, and it promotes the formation of controllable, uniform cracks during stretching (Figure S13a–e). This dual functionality enables the micropatterned composite hydrogel sheet to exhibit high sensitivity even under subtle strains (Figure 4d), along with excellent stability and durability—particularly at low strains, as illustrated in Figure 4e.

For the stability and durability characterization, while 24-h continuous testing is ideal, its implementation is limited by the extensive platform resource occupation—thus, a time-efficient strategy using high-frequency loading-unloading cycles was adopted (Figure 4e and Figure S15). The strain sensor based on the micropatterned composite hydrogel sheet maintains stable and repeatable sensing performance throughout 800 loading and unloading cycles at 5% strain (Figure 4e), and remains relatively stable after over 450 cycles at 50% strain (Figure S14a). This stability stems from the strong MWCNT-hydrogel bonding and the formation of controllable, uniform cracks. In contrast, the sensor based on the non-micropatterned composite hydrogel sheet begins to exhibit unstable and irregular real-time ∆R/R_0_ after 400 cycles at 50% strain (Figure S14b), with this instability attributed to the formation of large, irregular cracks and MWCNT detachment. However, during the long-term use of the micropatterned composite hydrogel sensor, there may still be changes in electrical conductivity caused by the oxidation and detachment of MWCNTs, as well as mechanical property instability induced by hydrogel dehydration—both of which can lead to fluctuations in the sensor’s performance. Therefore, in future work, we will focus on systematic long-term degradation testing (including chemical stability, mechanical durability, and MWCNT network integrity) to further optimize the sensor’s lifespan for extended real-world applications.

Moreover, the micropatterned composite hydrogel sheet’s freezing resistance and high-temperature tolerance enable its based sensor based to operate effectively even after conditioning at −20 °C for 24 h or 60 °C for 2 h (Figure 4f). Specifically, after 24-h conditioning at −20 °C, the sensor exhibits ultra-high GF of 104.36 and 206.75 in the strain ranges of 0–30% and 30–100%, respectively. This enhanced GF at low temperature is attributed to the hydrogel’s increased stretchability and reduced tensile strength, which promotes deformation and crack formation in the MWCNT coating.

After conditioning at 60 °C for 2 h, the strain sensor’s ∆R/R_0_ versus strain curve exhibits three distinct linear segments across 0–25%, 25–60%, and 60–100% strain (Figure 4f), with corresponding GF of 66.5, 198.8, and 635, respectively. The reduced GF at relatively low strains and enhanced GF at higher strains arise from water evaporation in the micropatterned composite hydrogel sheet, which causes 37.17% water loss. This water loss induces shrinkage and structural changes in both the hydrogel matrix and MWCNT coating: the hydrogel hardens (Figure 3i) due to network densification from shrinkage [45], while the rigid, thick MWCNT coating forms wrinkles or folds [46]—particularly at geometric transition zones of protrusions and depressions, where localized hydrogel compression occurs. During stretching, the unfolding of these MWCNT wrinkles/folds mitigates resistance changes at low strains. In contrast, hydrogel stiffening intensifies stress concentration at geometric transitions, promoting more extensive/larger crack formation at higher strains and thus amplifying resistance changes.

As anticipated, the strain sensor based on the micropatterned composite hydrogel sheet exhibits excellent stability and durability under extreme temperature conditions, as demonstrated in Figure 4g,h and Figure S16a,b. After conditioning at −20 °C for 24 h (freezing) or 60 °C for 2 h (heating), the sensor maintains stable output signals throughout 400 loading-unloading cycles at 5% strain (Figure 4g,h); even when subjected to over 450 cycles at 50% strain, only a slight signal upshift is observed (Figure S15a,b).

Additionally, the sensor based on the micropatterned composite hydrogel sheet retains good sensing performance under sweaty conditions—simulated by coating a continuous layer of artificial sweat on its MWCNT-free side (Figure S16)—owing to the highly crosslinked structure of the PVA-PAA-Zr^4+^ hydrogel matrix. As shown in Figure S16a, it maintains high GF of 37.74, 70.73, and 190.77 across 0–30%, 30–60%, and 60–100% strain ranges, respectively—values significantly higher than those of the same hydrogel-based strain sensors without sweat exposure (Table S2). Even in the small strain range of 0.1–0.9% (Figure S16b), the sweat-exposed sensor still outputs high, repeatable ∆R/R_0_ values proportional to applied strain; at the 0.1% detection limit, ∆R/R_0_ remains above 1%. For 1% applied strain (Figure S16c), the response and recovery times increase lightly to 340 ms and 330 ms, respectively—moderate values even compared to the sweat-free hydrogel-based strain sensor (Table S2).

### 2.4. Function Analysis of Surface Micropattern

The establishment of a continuous MWCNT conductive network on the surface of the PVA-PAA-Zr^4+^ hydrogel sheet—with the hydrogel prevented from penetrating the network—enables the fabrication of a composite hydrogel sheet with high conductivity and excellent sensing properties. However, the rigidity mismatch between the stiff MWCNT coating and the soft PVA-PAA-Zr^4+^ hydrogel matrix, combined with interfacial weak adhesion, renders the MWCNT coating prone to cracking, delamination, and detachment during stretching. As shown in Figure S14a–c, the non-micropatterned composite hydrogel sheet forms uncontrollable large cracks and even peels off during stretching—this defect makes it unable to withstand repeated loading-unloading even at small tensile strains (e.g., 5%, Figure S14b).

Incorporating a reverse sandpaper micropattern onto the PVA-PAA-Zr^4+^ hydrogel surface enhances MWCNT anchoring through multiple synergistic effects: the rough micropattern significantly increases the active surface area, exposing more functional groups for interaction with the MWCNTs. Simultaneously, the irregular protrusions and depressions on the hydrogel surface improve wettability to MWCNTs and facilitate mechanical interlocking between the MWCNT coating and hydrogel matrix. Compared to the non-micropatterned hydrogel sheet, these features enable more effective anchoring of MWCNTs on the micropatterned surface, thereby mitigating MWCNT delamination and detachment during stretching.

**Figure 5:**
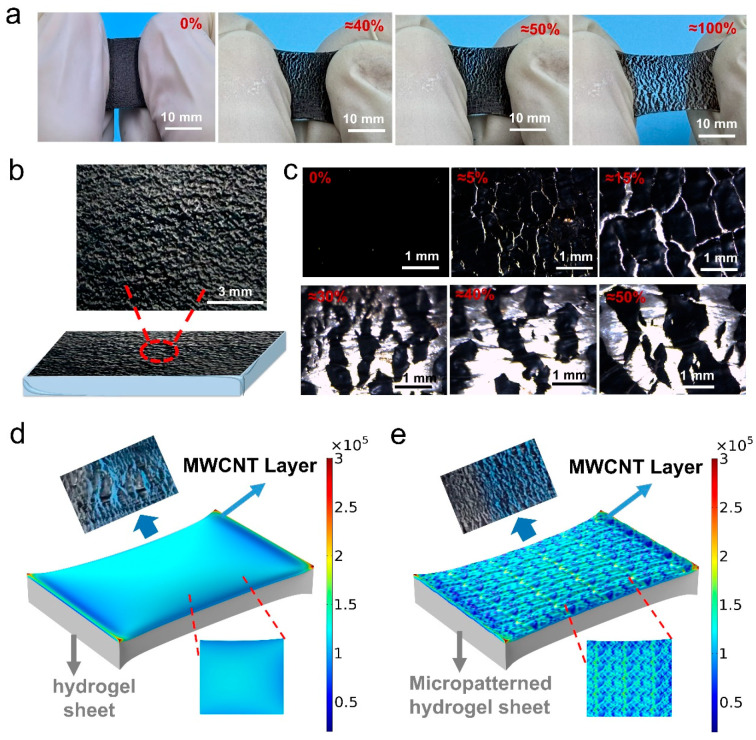
(**a**) Photographs of the stretched micropatterned composite hydrogel sheet, showing its deformation under different strains. (**b**) Schematic illustration of the layered structure of the micropatterned composite hydrogel sheet. (**c**) Optical microscope images of the stretched micropatterned composite hydrogel sheet, demonstrating crack formation in its MWCNT layer under different strains. Simulation of deformation and stress distribution in (**d**) the non-micropatterned composite hydrogel sheet and (**e**) the micropatterned composite hydrogel sheet at 50% strain.

Moreover, as shown in Figure 5a, the MWCNT coating on the micropatterned composite hydrogel sheet (Figure 5b) forms relatively regular, fine cracks during stretching—these cracks progressively enlarge with increasing applied strain but remain firmly bonded to the hydrogel matrix. Optical microscopy images (Figure 5c) further reveal that the MWCNT coating generates observable, uniformly distributed microcracks even at a small 5% strain, with maximum width ranging from 8.47 to 38.14 µm and an average of 21.35 µm (Table S3). Notably, the cracks initiate at the geometric transitions of the hydrogel’s protrusions and depressions and expand with increasing tensile strain, resulting in a fragmented but interconnected MWCNT network (Figure 5c); for example, as strain increases to 15%, 30%, 40%, and 50%, the average maximum crack width rises significantly to 63.43, 204.10, 466.86, and 515.07 µm, respectively (Table S3). This controlled cracking behavior significantly enhances the resistance change in the micropatterned composite hydrogel sheet at small strains and enables the MWCNT coating to produce predictable cracks and resistance changes even at relatively high strains, endowing the sensor with ultra-high sensitivity (even in the small strain range) as well as excellent durability and stability.

To explain the uniform, controllable crack formation of the MWCNT coating on the micropatterned composite hydrogel sheet during stretching, finite element simulation analyses were conducted on both micropatterned and non-micropatterned composite hydrogel sheets. As shown in Figure 5d and Figure S17a,b, the non-micropatterned composite hydrogel sheet exhibits no obvious stress concentration in most regions when stretched to 50% strain—only slightly higher stress in the central area and significant stress concentration at the four corners and edges (caused by abrupt geometric shape changes). This uneven stress distribution leads to randomly generated cracks and the formation of large cracks, which originate from weak interfacial bonding, mismatched tensile properties, and inherent defects in the hydrogel-supported MWCNT coating.

In contrast, for the micropatterned composite hydrogel sheet (Figure 5e and Figure S17c,d), stress concentration is primarily localized at the geometric transitions of the protrusions and depressions—consistent with the positions where cracks initiate during stretching (Figure 5c). This indicates that the formation of small and uniformly distributed cracks in the MWCNT coating is predominantly driven by the micropattern-induced stress concentration and the uniform spatial distribution of the hydrogel’s protrusions and depressions. The localized, amplified force from this stress concentration promotes microcrack formation in the MWCNT coating even under small strains, leveraging the tensile property mismatch between the MWCNT coating and the hydrogel matrix. Additionally, the enhanced bonding strength between the MWCNT coating and the hydrogel enables the formation of controllable, tailorable cracks even at large strains. These synergistic features endow the micropatterned composite hydrogel sheet with ultra-high sensitivity in the small strain range, along with excellent stability and durability at larger strains.

In summary, this simulation reveals that the micropatterns induce localized stress concentration, which promotes the formation of controllable microcracks in the MWCNT conductive network during stretching. This stepwise microcrack propagation amplifies the resistance response to strain, directly boosting GF and enabling the micropatterned composite hydrogel sensor to achieve a superior GF compared to most reported CNT-based hydrogel sensors (Table S2). Meanwhile, the strong interfacial bonding between MWCNTs and the hydrogel matrix ensures the stability of the conductive network, enabling reproducible sensing performance.

### 2.5. Applications

Biocompatibility is critical for the practical application of skin-contact sensors. The core components of our sensor—PVA, PAA, glycerol, MWCNTs, and ZrCl_4_—are widely recognized as biocompatible materials with established safety profiles for skin-contact use. PVA, PAA, and glycerol are commonly employed in biomedical materials such as wound dressings due to their non-toxicity and excellent skin compatibility. Meanwhile, MWCNTs with the specified dimensions (<10 μm length, >50 nm diameter) have been shown to exhibit minimal cytotoxicity in skin-contact scenarios in numerous prior studies [47,48,49]. For Zr^4+^, its low toxicity and biocompatibility are well-documented [50], and the 0.82 wt% ZrCl_4_ loading in our hydrogel is far below concentrations associated with skin irritation. Thus, the micropatterned composite hydrogel sensor is safe for repeated skin application.

**Figure 6:**
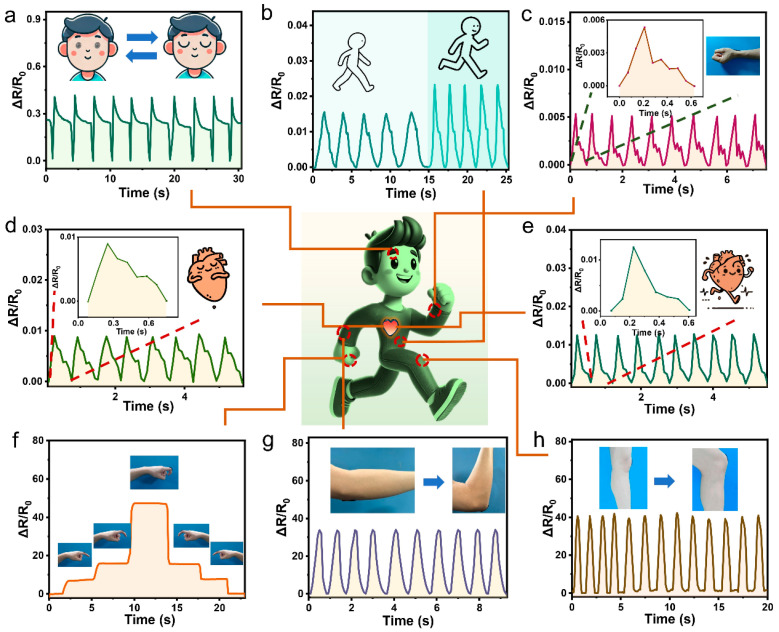
(**a**) Real-time ∆R/R_0_ of the sensor based on the micropatterned composite hydrogel sheet for blinking detection (attached to the upper eyelid). (**b**) Real-time ∆R/R_0_ for respiratory monitoring (attached to the abdomen). (**c**) Real-time ∆R/R_0_ for pulse wave recording (attached to the wrist). (**d**) Real-time ∆R/R_0_ for resting heartbeat monitoring (attached to the cardiac region) (**e**) Real-time ∆R/R_0_ for post-exercise heartbeat monitoring (attached to the cardiac region). (**f**) Real-time ∆R/R_0_ for finger bending detection (attached to the index finger). (**g**) Real-time ∆R/R_0_ for elbow bending detection (attached to elbow joint). (**h**) Real-time ∆R/R_0_ for knee bending detection during walking (attached to the knee joint of a volunteer).

The strain sensor based on the micropatterned composite hydrogel sheet was first applied to human activity monitoring: it was attached to different body parts, and real-time electrical signals induced by corresponding movements were recorded. As shown in Figure 6a and Figure S18a,b, when the sensor is affixed to various facial positions (e.g., superior eyelid, glabella, cheek) and subjected to facial expressions, it generates strong, repetitive electrical signals with distinct shapes and peak values. This demonstrates the sensor’s ability to accurately detect and distinguish specific facial expressions—including blinking, frowning, and puffing—via the production of expression-specific electrical signals.

Swallowing—a dynamic, continuous physiological movement—induces a series of intense, radial peristaltic motions of the Adam’s apple (Figure S18c), which can be accurately detected by attaching the sensor to a volunteer’s throat. Meanwhile, the respiratory process causes subtle undulating changes in the abdomen: when the micropatterned composite hydrogel sensor is affixed to the abdomen of a male volunteer, it enables real-time monitoring of respiratory frequency, depth, and whether respiration is obstructed during walking or slow running (Figure 6b), providing valuable support for the diagnosis of respiratory system diseases.

Owing to its high sensitivity and ultra-low strain detection limit, the strain sensor based on the micropatterned composite hydrogel sheet is capable of distinguishing subtle physiological activities. As illustrated in Figure 6c, when affixed to a volunteer’s wrist, the sensor can monitor the volunteer’s health status by detecting periodic waveforms with specific frequencies—these waveforms exhibit three characteristic peaks: the percussion wave (P-wave), tidal wave (T-wave), and dichrotic wave (D-wave) [51].

Similarly, attaching the sensor to a volunteer’s cardiac region enables heart activity monitoring. As illustrated in Figure 6d, when the volunteer is at rest, the sensor outputs periodic electrical signals corresponding to a heart rate of 84 beats per minute, with waveforms featuring three distinct peaks. Notably, after the volunteer completes exercise (Figure 6e), the sensor records periodic signals with an elevated heart rate of 109 beats per minute—these signals exhibit enhanced amplitude and altered waveforms characteristics, which reflect increased cardiac contractility, accelerated blood flow, heightened metabolic activity, and intensified chest cavity expansion/contraction.

Human joint bending typically induces relatively larger strains compared to other physiological activities. As shown in Figure 6f, when the sensor is attached to the index finger joint, the amplitude of its electrical signal progressively increases as the finger bends from 0° to 30°, 45°, and ultimately 90°; conversely, the signal stepwise recovers to its original level as the finger extends back from 90° to 45°, 30°, and 0°. This observation confirms a direct correlation between the sensor’s electrical signal amplitude and the finger’s bending angle.

When attached to the wrist, elbow, and knee (Figure S18d and Figure 6g,h), the sensor generates regular, periodic electrical signals during repeated wrist/elbow bending and normal walking—with signal amplitude progressively increasing in line with the increasing size of the wrist, elbow, and knee joints [9]. This confirms that the micropatterned composite hydrogel-based sensor can effectively function as a wearable sensor for monitoring diverse human activities, health status, and physical conditions. Additionally, the sensor exhibits robust stability for human activity monitoring (e.g., finger bending, Figure S19): when exposed to air for 0–6 days and coated with artificial sweat on its non-patterned surface, ΔR/R_0_ fluctuations (attributed to hydrogel dehydration) remain within ±20%, and even after 6 consecutive days of air exposure and in the presence of artificial sweat, it consistently outputs stable, repeatable electrical signals without observable baseline shifts caused by degraded skin adhesion (Figure S19).

**Figure 7:**
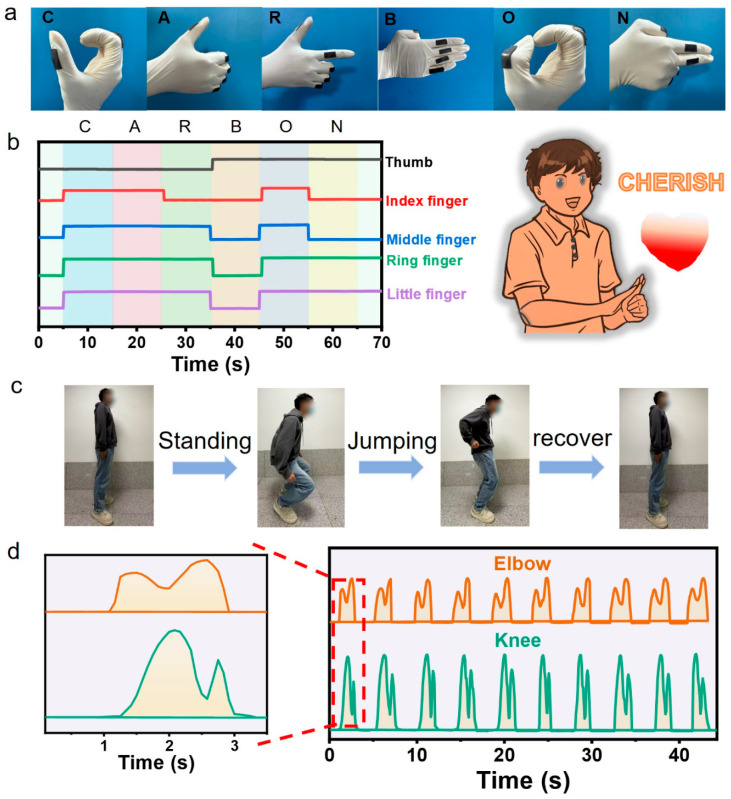
(**a**) Letters represented by different sign language gestures. (**b**) Real-time ∆R/R_0_ of the micropatterned composite hydrogel sheet-based sensors attached to the fingers during the performance of sign language for “CARBON.” (**c**) Decomposition of the standing long jump movement. (**d**) Real-time ∆R/R_0_ of the sensors attached to the knees and elbows joints for monitoring the standing long jump.

In addition, the combination of multiple sensors enables the capture of more complex human activities. As illustrated in Figure 7a,b, five individual sensors attached to different finger joints can simultaneously detect each finger’s movement and position. When sequential gestures corresponding to the letters C, A, R, B, O, and N are performed, the sensors collectively convey the word “CARBON” via real-time electrical signals generated for each gesture. This functionality can assist individuals with oral communication difficulties, enabling more effective communication and addressing the challenges posed by speech impairment.

As shown in Figure 7c, attaching the sensors to a volunteer’s elbow and knee joints enables simultaneous detection of their movements during a standing long jump, with the sensors outputting real-time electrical signals of the same frequency but distinct waveforms. Specifically, during the jump, the arms are swung forward before takeoff and folded in the latter phase, while the thigh bend to maximum extent to store energy pre-jump and remain stationary post-landing. Accordingly, the elbow and knee sensors generate periodic electrical signals featuring one strong peak and one weak peak (Figure 7d). Notably, the weak peak of the elbow sensor’s signal precedes the strong peak, whereas the knee sensor’s signal exhibits the opposite order, and the peak positions of the knee sensor’s signal are staggered relative to those of the elbow sensor—indicating the alternating bending and straightening of the elbow and knee joints.

Performing multiple consecutive jumps allows for analysis of jump patterns, and the jump distance can be assessed based on the amplitude and characteristic features of the electrical signals (Figure 7d). This capability enables application in precise motion analysis, providing valuable insights to optimize athletic performance.

## 3. Conclusions

In summary, a micropatterned composite hydrogel sheet was fabricated by coating a layer of MWCNTs on a micropatterned PVA-PAA-Zr^4+^ hydrogel sheet—where the latter was prepared via a two-step gelatinization process using sandpaper as a template. The excellent conductivity of MWCNTs enables the formation of a continuous electron-conductive network, endowing the composite hydrogel sheet with exceptional conductivity and strain sensing performance, while the micropatterned PVA-PAA-Zr^4+^ hydrogel matrix imparts superior mechanical properties, antibacterial properties, permeability, self-healing capability, and adhesion. The hydrogel’s surface micropattern significantly enhances the stability and durability of the composite as a strain-sensing material. Additionally, the incorporation of glycerol and the hydrogel’s highly crosslinked structure enable the sensor to operate effectively at extreme temperatures (−20 °C and 60 °C) and under sweaty conditions. The resulting strain sensor exhibits ultra-high GF of 76.1 (0–30% strain) and 203.5 (30–100% strain), an extremely low strain detection limit of 0.1%, excellent stability and durability (particularly at low strains), and balanced response/recovery time of 250/250 ms—making it well-suited for human activity monitoring and health assessment. Furthermore, the simultaneous operation of multiple sensors expands the composite hydrogel’s potential applications to more complex scenarios, including language expression, communication assistance, motion analysis, and athletic performance optimization. This study thus provides a promising research direction for the development of ultra-sensitive conductive hydrogel-based sensors.

## 4. Materials and Methods

### 4.1. Materials

Acrylic acid (AA) was purchased from Tianjin Damao Chemical Reagent Factory (Tianjin, China). Ammonium persulfate (APS) was obtained from Sinopharm Chemical Reagent Co., Ltd. (Shanghai, China). Low-viscosity polyvinyl alcohol (PVA1788, alcoholysis degree 87–89%, relative molecular weight ~22,000), zirconium tetrachloride (ZrCl_4_), and N,N,N′,N′-tetramethylenediamine (TEMED) were sourced from Shanghai McLean Biochemistry Co., Ltd. (Shanghai, China). Multi-walled CNTs (MWCNTs)—with a length of <10 μm and diameter of >50 nm—were acquired as a 14 wt% aqueous dispersion from Nanjing Pioneer Nanotechnology Co., Ltd. (Nanjing, China). Glycerol was purchased from Tianjin Fuyu Fine Chemical Co., Ltd. (Tianjin, China).

### 4.2. Preparation of Micropatterned Composite Hydrogel Sheet

The PVA-PAA-Zr^4+^ hydrogel precursor was prepared by mixing 7 g of a 10 wt% PVA aqueous solution, 3 g of glycerol, 2 g of AA, and 0.1 g of ZrCl_4_, followed by the addition of 44 mg of APS and 40 μL of TEMED. A pipette was used to transfer 1.5 mL of the precursor into a polytetrafluoroethylene (PTFE) mold with dimensions of 40 mm × 20 mm × 5 mm. The PTFE mold was covered with a thin glass sheet and placed in an oven at 60 °C for 1.5 h. Afterward, sandpaper (120 mesh) with an arithmetic mean roughness of 24.47 µm (Figure S20) was placed on top of the pre-formed hydrogel; the mold was covered again with the glass sheet and returned to the oven at 60 °C for an additional 1 h. It should be noted that the PVA-to-AA mass ratio and crosslinking density—specifically, the ZrCl_4_-to-AA mass ratio in the hydrogel precursor—were optimized in advance based on two key criteria: whether the gelatinized hydrogel sheet could be easily peeled from the sandpaper template while maintaining an intact reverse pattern, and whether the micropatterned hydrogel sheet exhibited adequate stretchability. Table S1 lists the formulations used in the optimization process of the PVA-PAA-Zr^4+^ hydrogel precursor. Furthermore, the mesh count of the sandpaper template was optimized considering several factors: ease of peeling, the hydrogel’s ability to replicate a perfect micropattern, the stability of the MWCNT coating on the micropatterned hydrogel surface, and the impact of the MWCNT coating on the hydrogel’s morphology. The optimization process of the sandpaper template is summarized in Table S4. The resulting hydrogel sheet, with a reverse sandpaper micropattern, was removed from the mold and coated with an MWCNT aqueous dispersion to form a bumpy, continuous MWCNT layer on its surface. For comparison, flat composite hydrogel sheets and micropatterned hydrogel sheets without the MWCNT coating were also prepared under the same conditions. To prevent evaporation-induced water loss, all hydrogel sheets were placed in self-sealing polypropylene pouches and stored at 4 °C. For transportation of the micropatterned composite hydrogel sheets, a multi-layer packaging strategy as outlined in Table S5 is recommended.

### 4.3. Characterization

The morphology of the micropatterned composite hydrogel sheet was characterized using a scanning electron microscopy (SEM, Hitachi Regulus 8220, Hitachi High-Tech Corp., Tokyo, Japan). The chemical composition and valence states of the PVA-PAA-Zr^4+^ hydrogel sheet surface were analyzed via X-ray photoelectron spectroscopy (XPS, ESCALAB Xi+, Thermo Fisher Scientific, Waltham, MA, USA). The functional groups of acrylic acid and the PVA-PAA-Zr^4+^ hydrogel sheet were examined using an ALPHA infrared spectrometer (IR, ALPHA model, Bruker Optics, Ettlingen, Germany). All samples for SEM, XPS, and IR analyses were freeze-dried prior to testing. Tensile tests were performed on the micropatterned PVA-PAA-Zr^4+^ hydrogel sheet and micropatterned composite hydrogel sheet (40 mm × 20 mm × 1.2 mm) using a texture analyzer (TA, XT Plus C, Stable Micro Systems Ltd., Guildford, UK), with stress–strain curves acquired at a tensile speed of 40 mm/min. The same texture analyzer was employed to evaluate the adhesion of the PVA-PAA-Zr^4+^ hydrogel sheet to various substrates via the lap shear test method (sample size: 20 mm × 20 mm × 1.2 mm), following the protocol reported previously [25]. For sensing performance evaluation, the micropatterned hydrogel sheet and micropatterned composite hydrogel sheet (20 mm × 20 mm × 1.2 mm) were subjected to varying strains using the texture analyzer, while their real-time resistance was recorded. Resistance measurements were conducted with a digital source meter (Model 2450, Keithley Co., Solon, OH, USA), in accordance with the methodology described in our previous work [52]. All tests involving quantitative data with potential variability—including stress–strain tests, adhesion strength measurements, mechanical self-healing performance evaluations, and relative resistance change (∆R/R_0_) tests—were repeated at least three times to ensure experimental reproducibility.

### 4.4. Finite Element Simulation Analysis

The deformation and stress distribution of the composite hydrogel sheet and micropatterned composite hydrogel sheet during stretching were simulated using the Structural Mechanics module of COMSOL Multiphysics^®^ version 6.0 (COMSOL Inc., Burlington, MA, USA). To simplify the simulation, geometric models were constructed as two-layer composite sheets, where both the hydrogel matrix and the MWCNT coating were treated as isotropic materials with distinct mechanical properties. The micropattern was modeled as a series of repetitively arranged irregular protrusions. For the hydrogel sheet, the elastic modulus was set to 86 kPa and Poisson’s ratio to 0.35; for the MWCNT coating, the elastic modulus was set to 211 kPa and Poisson’s ratio to 0.15. The micropattern was simulated under the Structural Mechanics physical field, with initial displacement and initial velocity both set to 0. Boundary conditions were defined as follows: one side was fixed, while the other side was assigned a specified displacement to simulate the material’s deformation and mechanical response under tension.

## Data Availability

The original contributions presented in this study are included in the article/Supplementary Materials. Further inquiries can be directed to the corresponding author.

## Supplementary Materials

The following supporting information can be downloaded at: https://www.mdpi.com/article/10.3390/gels11110913/s1, Figure S1. Photographs of (a) sandpaper, (b) micropatterned PVA-PAA-Zr^4+^ hydrogel sheet, and (c) micropatterned composite hydrogel sheet at different magnifications; Figure S2. (a) O 1s XPS spectrum and (b) Zr 3d XPS spectrum of PVA-PAA-Zr^4+^ hydrogel sheet. (c) XRD pattern, (d) Raman spectrum of MWCNTs; Figure S3. Energy storage modulus (G′) and loss modulus (G″) of (a) PVA-PAA-Zr^4+^ hydrogel sheet and (b) composite hydrogel sheet at alternating step strains between 1% and 1100% measured at a frequency of 6.28 rad/s; Figure S4. (a) Stress–strain curves of PVA-PAA-Zr^4+^ hydrogel sheet and composite hydrogel sheet without surface micropattern. (b) Young’s modulus of PVA-PAA-Zr^4+^ hydrogel sheet, micropatterned hydrogel sheet, composite hydrogel sheet and micropatterned composite hydrogel sheet; Figure S5. Photographs of (a) micropatterned composite hydrogel sheet and (b,c) stretched micropatterned composite hydrogel sheet; Figure S6. Elongation at break and toughness of PVA-PAA-Zr^4+^ hydrogel sheets and composite hydrogel sheets with and without micropattern; Figure S7. Stress–strain curves of cut micropatterned composite hydrogel sheet (a) after directly butt-jointed at room temperature for different time, (b) after coated with glycerol-water binary solvent and butt-jointed for different durations at room temperature, (c) after coated with glycerol-water binary solvent and butt-jointed for 1.5 h at 60 °C, and (d) after coated with hydrogel precursor and butt-jointed for 1.5 h at 60 °C; Figure S8. Water loss curve of micropatterned composite hydrogel sheet; Figure S9. Photographs of micropatterned hydrogel sheet for showing its adhesion to different materials; Figure S10. Variation in water permeability of micropatterned composite hydrogel sheet; Figure S11. Inhibition effect of (a,d) PVA-PAA hydrogel sheet, (b,e) PVA-PAA-Zr^4+^ hydrogel sheet and (c,f) micropatterned composite hydrogel sheet on (a–c) *Escherichia coli* and (d–f) *Staphylococcus aureus*; Figure S12. ∆R/R_0_ versus strain curves of strain sensors based on flat composite hydrogel sheet and flat PVA-PAA-Zr^4+^ hydrogel sheet and enlarged ∆R/R_0_ versus strain curve of PVA-PAA-Zr^4+^ hydrogel sheet-based strain sensor; Figure S13. Photographs of stretched (a–c) flat composite hydrogel sheet and (d,e) micropatterned composite hydrogel sheet for showing their crack production in MWCNT layer; Figure S14. Real-time ∆R/R_0_ of the strain sensor based on (a) micropatterned composite hydrogel sheet under a cyclic strain of 50% for 800 cycles, and (b) flat composite hydrogel sheet under a cyclic strain of 5% for 465 cycles at room temperature; Figure S15. Real-time ∆R/R_0_ of the micropatterned composite hydrogel sheet-based strain sensor under cyclic strain of 50% for 800 cycles after conditioning (a) at −20 °C for 24 h and (b) at 60 °C for 2 h; Figure S16. (a) ∆R/R_0_ versus strain curves of the sensor based on micropatterned composite hydrogel sheet coated with a continuous layer of artificial sweat. Real-time ∆R/R_0_ of the sensor based on the micropatterned composite hydrogel sheet coated with a continuous layer of artificial sweat (b) in the low strain range (0.1–0.9%) and (c) at 1% strain, with enlarged waveforms for showing the response and recovery time. * The artificial sweat (pH 5.5) was a Macklin product (CAS: NONE18743) manufactured by Shandong Keyuan Biochemical Co., Ltd.; Figure S17. Stress distribution of flat composite hydrogel sheet (a) in the length direction and (b) in width direction under simulated 50% strain. Stress distribution of micropatterned composite hydrogel sheet (c) in the length direction and (d) in width direction under simulated 50% strain; Figure S18. (a) Frowning detection by attaching the sensor between the eyebrows. (b) Puffing detection by attaching the sensor to the cheek. (c) Swallowing detection by attaching the sensor to throat. (d) Wrist bending detection by attaching the sensor to the wrist; Figure S19. Finger bending detection with sensor based on micropatterned composite hydrogel sheet after it exposed in air for (a) 0 days, (b) 2 days, (c) 4 days, and (d) 6 days and coated with artificial sweat; Figure S20. Profile and roughness curves of 120-mesh sandpaper template; Table S1. The optimization of PVA-PAA-Zr^4+^ hydrogel sheets; Table S2. Summary for performance of strain sensors based on conductive hydrogels containing carbon nanotubes; Table S3. The maximum width of cracks in MWCNT layer on micropatterned composite hydrogel sheet under different strains; Table S4. The optimization process of sandpaper template; Table S5: Multi-layer packaging strategy of micropatterned composite hydrogel sheet. References [26,53,54,55,56,57,58,59,60,61,62] are cited in the Supplementary Materials.
