# Supplementary material for: Micropatterned Composite Hydrogel Sheet with Surface Electronic Conductive Network for Ultrasensitive Strain Sensing
Source: Gels. 2025 Nov 15;11(11):913. doi: 10.3390/gels11110913 (PMC12652483; doi:10.3390/gels11110913)
Supplement: Supplementary file 1 [file gels-11-00913-s001.zip › gels-3967277-supplementary.pdf]

gels

Article

Micropatterned Composite Hydrogel Sheet with Surface Electronic Conductive Network for Ultrasensitive Strain Sensing

Ruidong Chu, Mingyu, Liu, Wenxia Liu *, Zhaoping Song, Guodong Li, Dehai Yu, Xiaona Liu, Huili Wang

State Key Laboratory of Green Papermaking and Resource Recycling, Qilu University of Technology, Shandong academy of science, Jinan 250353, China
* Corresponding to: liuwenxia@qlu.edu.cn

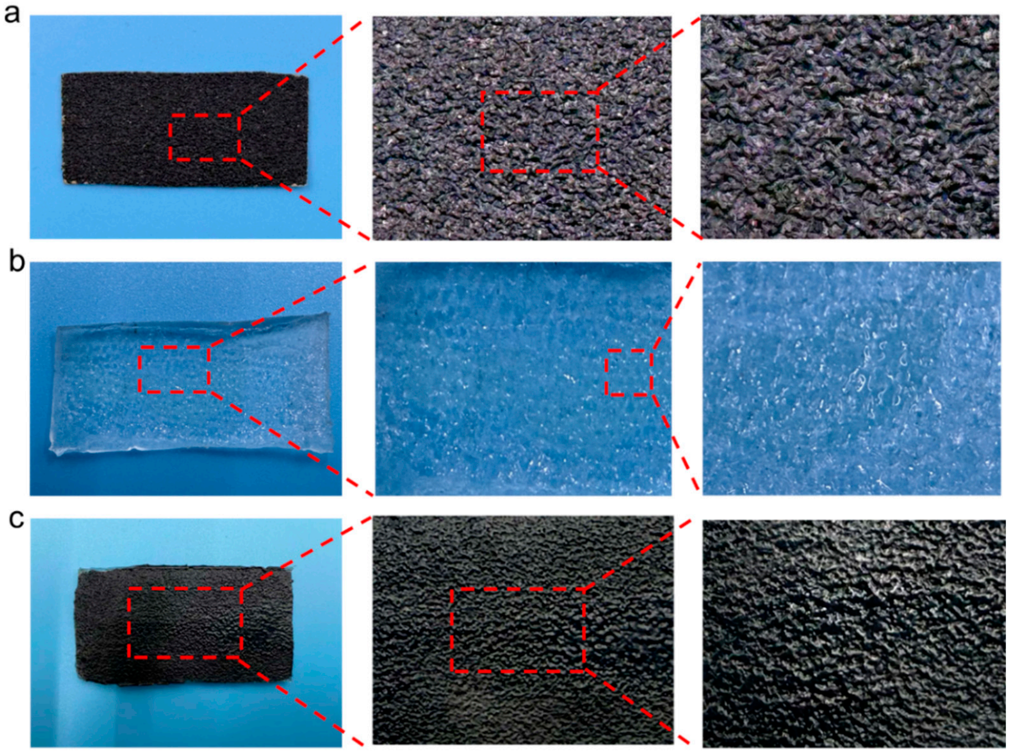

Figure S1. Photographs of (a) sandpaper, (b) micropatterned PVA-PAA-Zr^{4+} hydrogel sheet, and (c) micropatterned composite hydrogel sheet at different magnifications.

 https://doi.org/10.3390/gels11110913

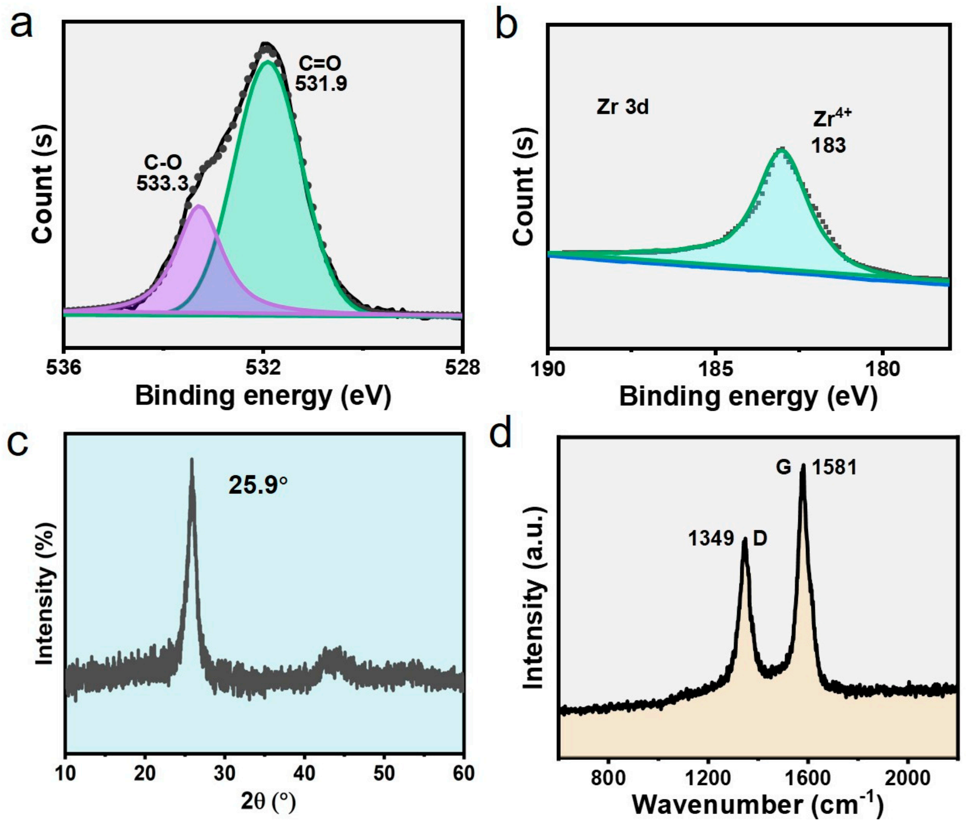

Figure S2. (a) O1s XPS spectrum and (b) Zr 3d XPS spectrum of PVA-PAA-Zr^{4+} hydrogel sheet. (c) XRD pattern, (d) Raman spectrum of MWCNTs.

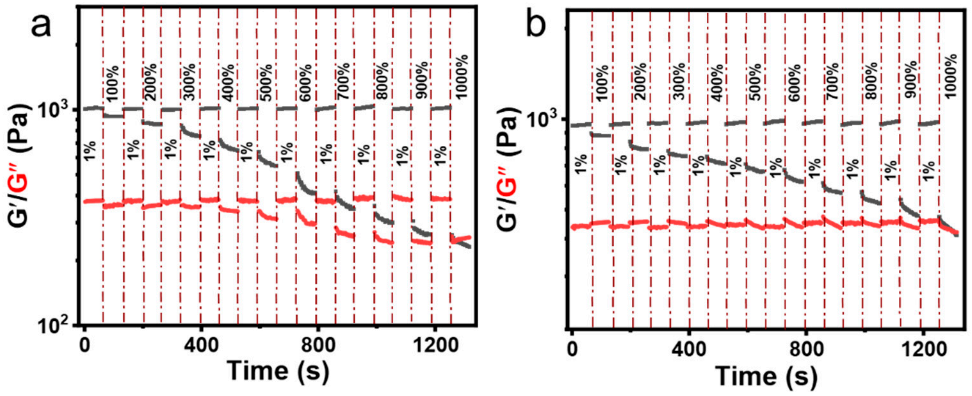

Figure S3. Energy storage modulus (G') and loss modulus (G") of (a) PVA-PAA-Zr^{4+} hydrogel sheet and (b) composite hydrogel sheet at alternating step strains between 1% and 1100% measured at a frequency of 6.28 rad/s.

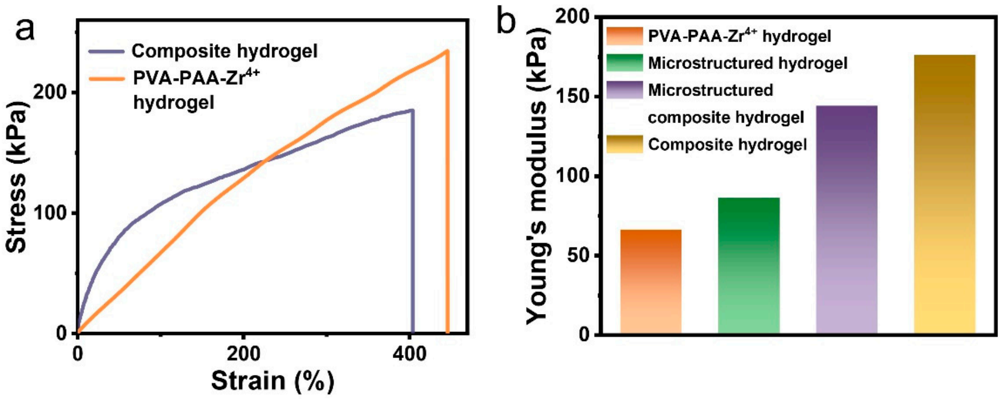

Figure S4. (a) Stress-strain curves of PVA-PAA-Zr^{4+} hydrogel sheet and composite hydrogel sheet without surface micropattern. (b) Young's modulus of PVA-PAA-Zr^{4+} hydrogel sheet, micropatterned hydrogel sheet, composite hydrogel sheet and micropatterned composite hydrogel sheet.

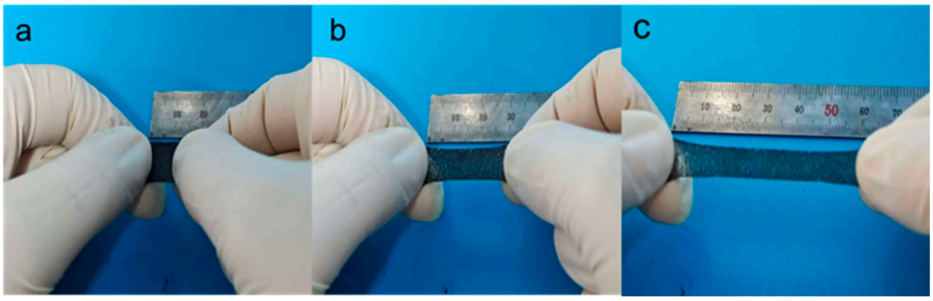

Figure S5. Photographs of (a) micropatterned composite hydrogel sheet and (b, c) stretched micropatterned composite hydrogel sheet.

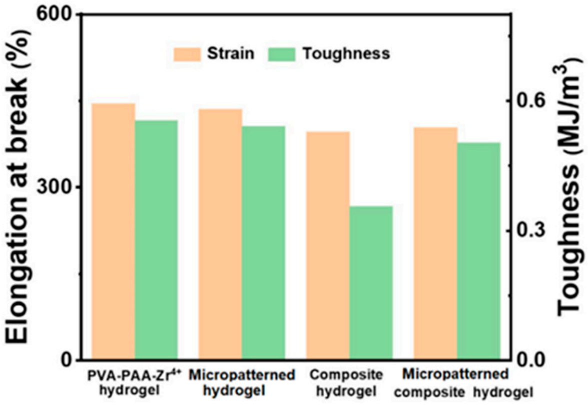

Figure S6. Elongation at break and toughness of PVA-PAA-Zr^{4+} hydrogel sheets and composite hydrogel sheets with and without micropattern.

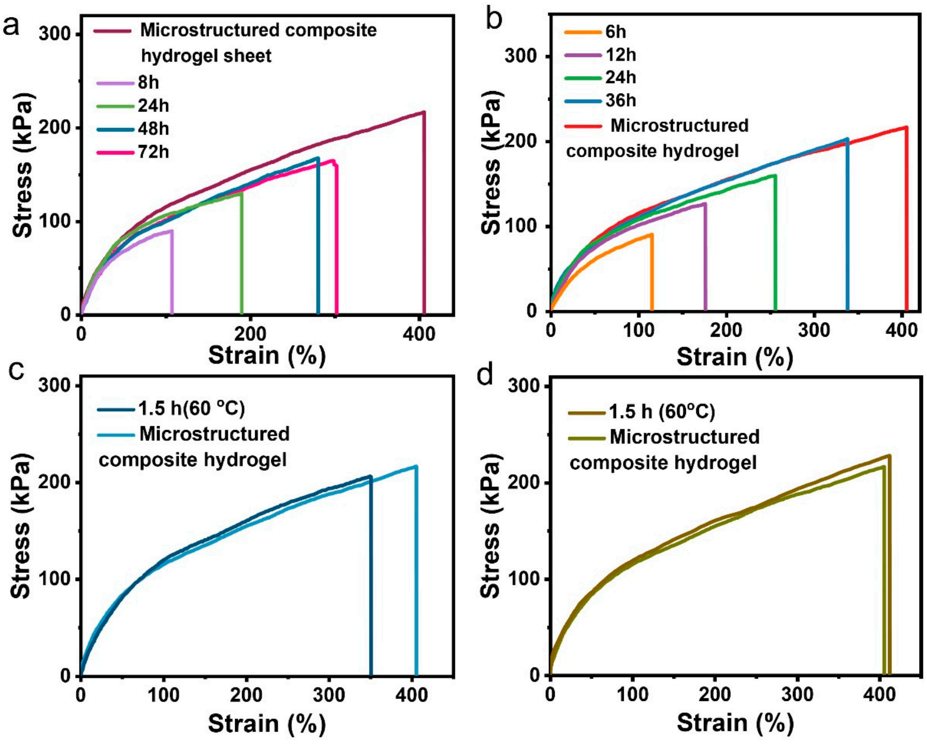

Figure S7. Stress-strain curves of cut micropatterned composite hydrogel sheet (a) after directly butt-jointed at room temperature for different time, (b) after coated with glycerol-water binary solvent and butt-jointed for different durations at room temperature, (c) after coated with glycerol-water binary solvent and butt-jointed for 1.5 hours at 60 °C, and (d) after coated with hydrogel precursor and butt-jointed for 1.5 hours at 60 °C.

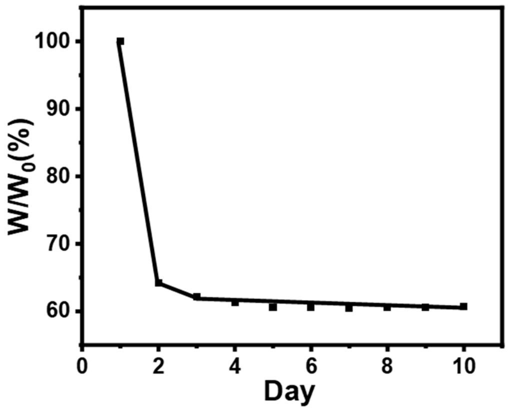

Figure S8. Water loss curve of micropatterned composite hydrogel sheet.

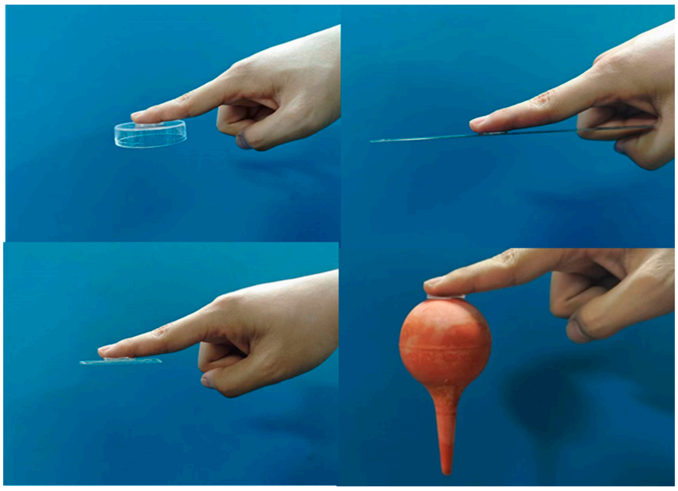

Figure S9. Photographs of micropatterned hydrogel sheet for showing its adhesion to different materials.

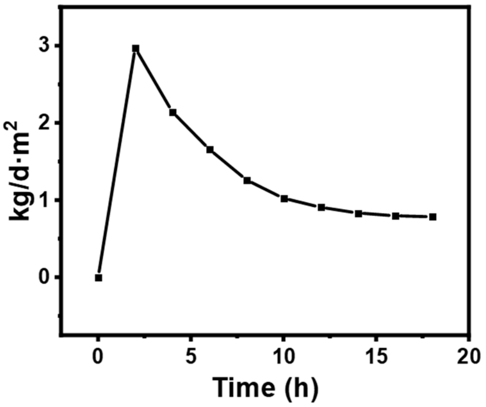

Figure S10. Variation in water permeability of micropatterned composite hydrogel sheet.

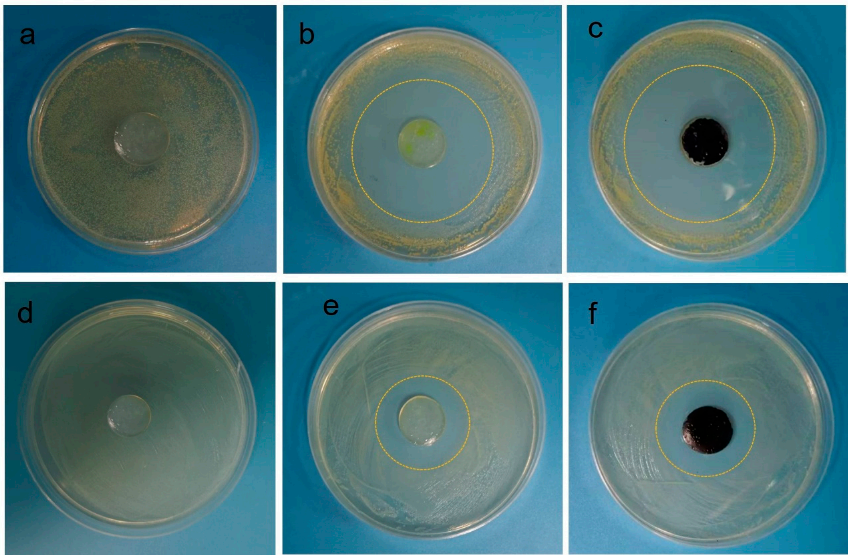

Figure S11. Inhibition effect of (a, d) PVA-PAA hydrogel sheet, (b, e) PVA-PAA-Zr^{4+} hydrogel sheet and (c, f) micropatterned composite hydrogel sheet on (a-c) Escherichia coli and (d-f) Staphylococcus aureus.

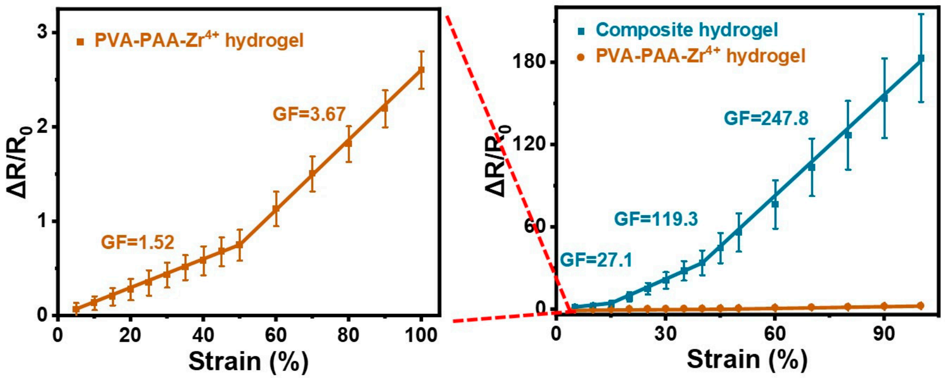

Figure S12. $\Delta R/R_0$ versus strain curves of strain sensors based on flat composite hydrogel sheet and flat PVA-PAA-Zr^{4+} hydrogel sheet and enlarged $\Delta R/R_0$ versus strain curve of PVA-PAA-Zr^{4+} hydrogel sheet-based strain sensor.

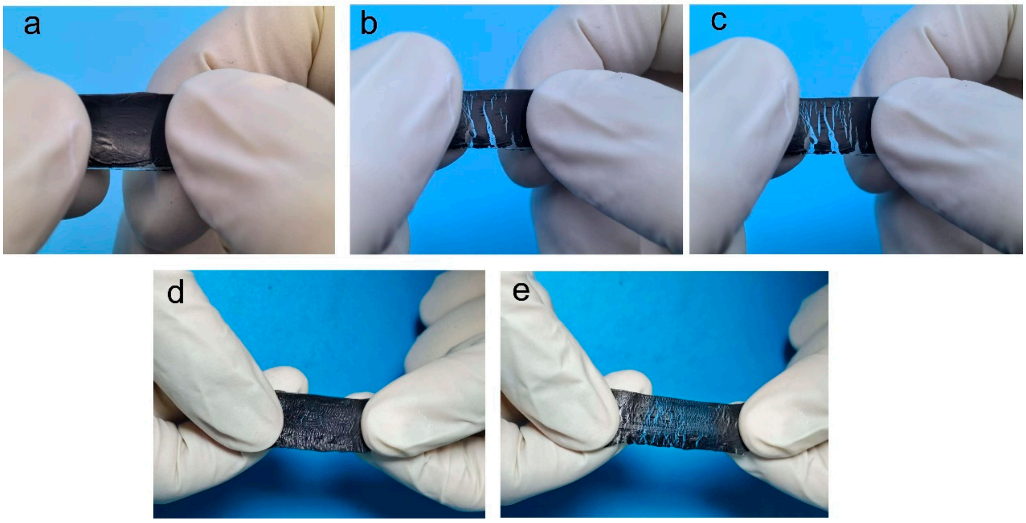

Figure S13. Photographs of stretched (a-c) flat composite hydrogel sheet and (d, e) micropatterned composite hydrogel sheet for showing their crack production in MWCNT layer.

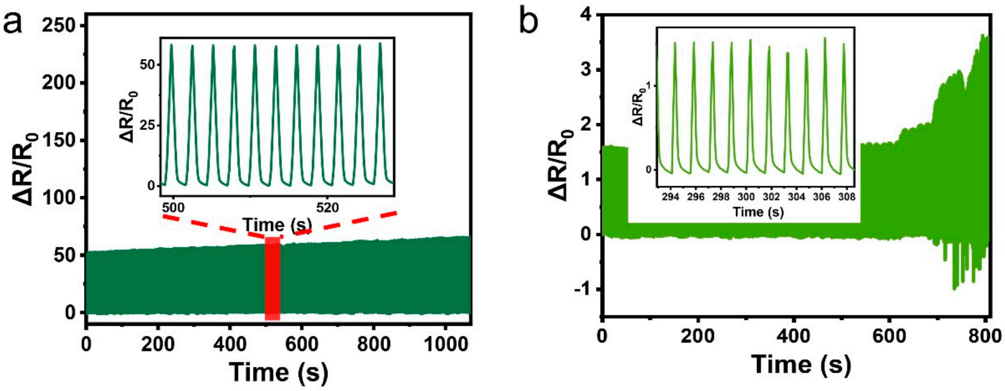

Figure S14. Real-time $\Delta R/R_0$ of the strain sensor based on (a) micropatterned composite hydrogel sheet under a cyclic strain of 50% for 800 cycles, and (b) flat composite hydrogel sheet under a cyclic strain of 5% for 465 cycles at room temperature.

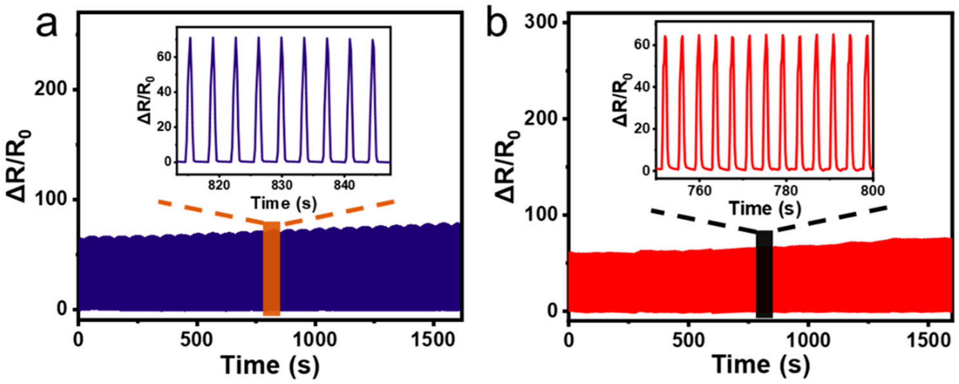

Figure S15. Real-time ΔR/R0 of the micropatterned composite hydrogel sheet-based strain sensor under cyclic strain of 50% for 800 cycles after conditioning (a) at -20°C for 24 hours and (b) at 60°C for 2 hours.

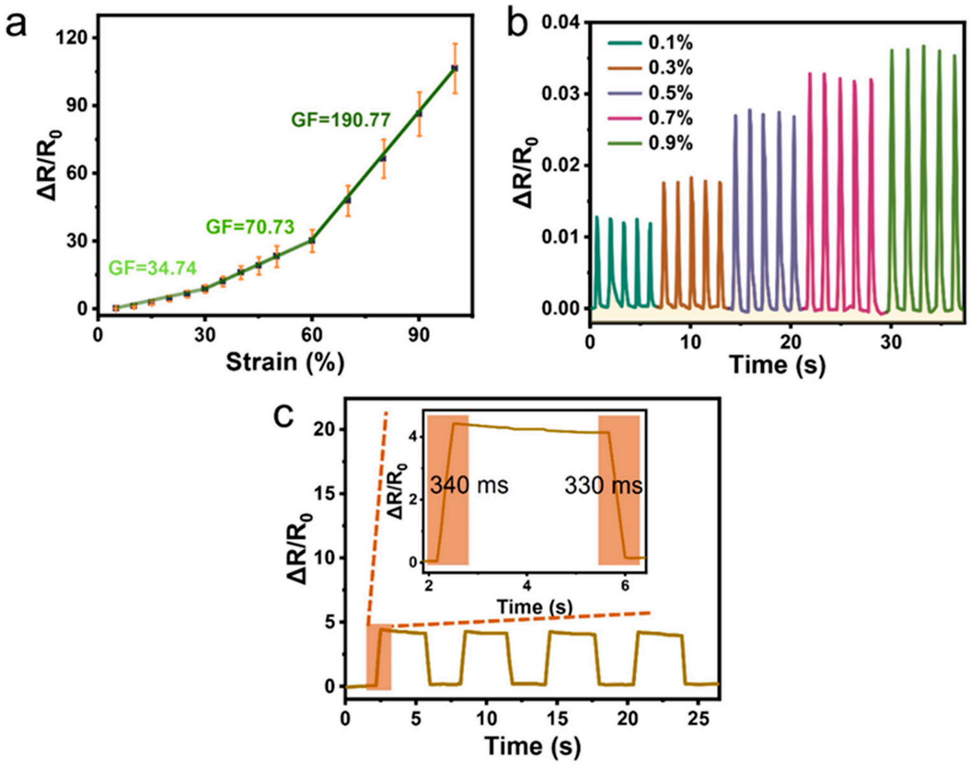

Figure S16. (a) ΔR/R0 versus strain curves of the sensor based on micropatterned composite hydrogel sheet coated with a continuous layer of artificial sweat. Real-time ΔR/R0 of the sensor based on the micropatterned composite hydrogel sheet coated with a continuous layer of artificial sweat (b) in the low strain range (0.1%-0.9%) and (c) at 1% strain, with enlarged waveforms for showing the response and recovery time.

* The artificial sweat (pH 5.5) was a Macklin product (CAS: NONE18743) manufactured by Shandong Keyuan Biochemical Co., Ltd.

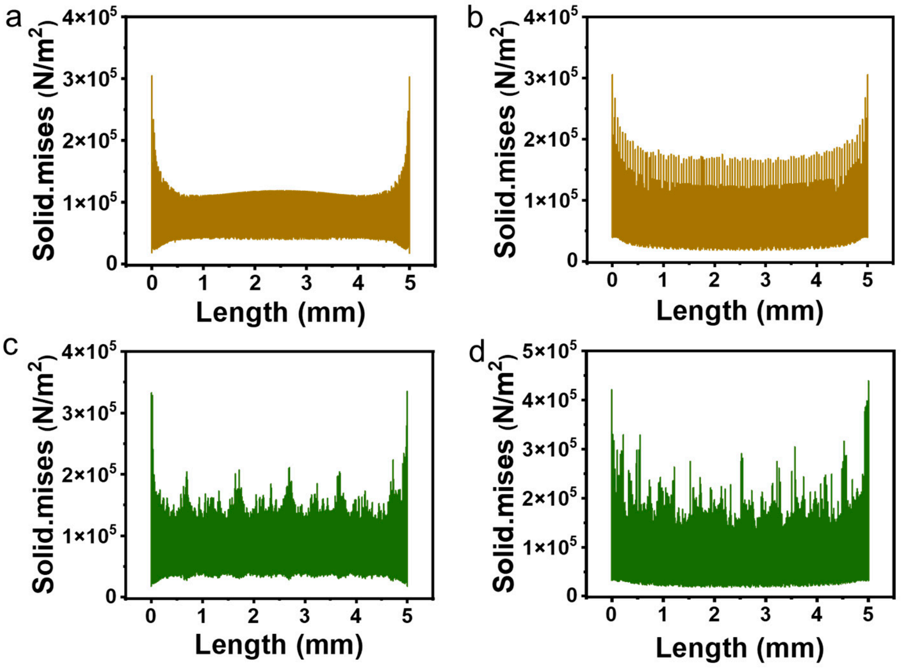

Figure S17. Stress distribution of flat composite hydrogel sheet (a) in the length direction and (b) in width direction under simulated 50% strain. Stress distribution of micropatterned composite hydrogel sheet (c) in the length direction and (d) in width direction under simulated 50% strain.

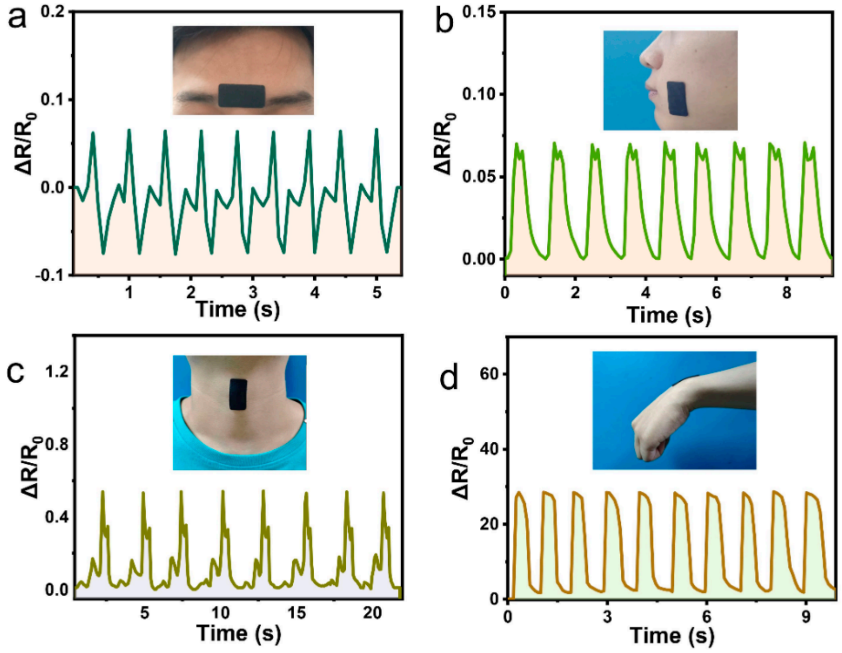

Figure S18. (a) Frowning detection by attaching the sensor between the eyebrows. (b) Puffing detection by attaching the sensor to the cheek. (c) Swallowing detection by attaching the sensor to throat. (d) Wrist bending detection by attaching the sensor to the wrist.

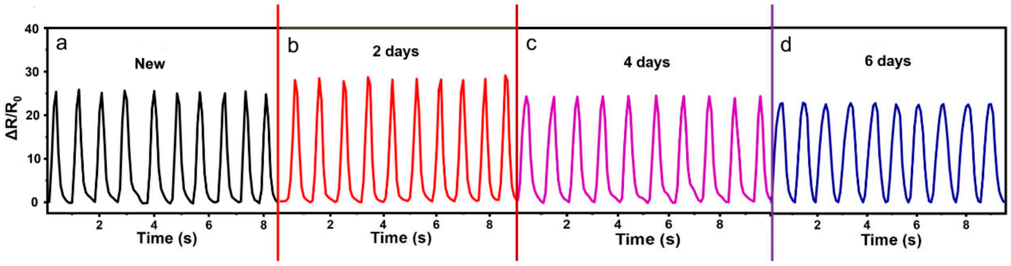

Figure S19. Finger bending detection with sensor based on micropatterned composite hydrogel sheet after it exposed in air for (a) 0 days, (b) 2 days, (c) 4 days, and (d) 6 days and coated with artificial sweat.

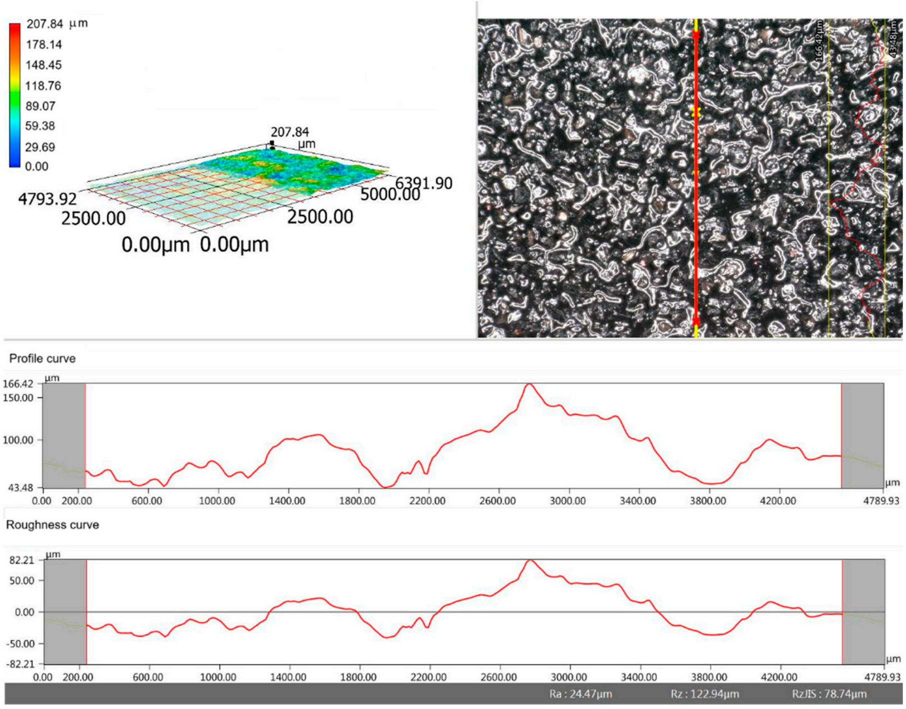

Figure S20. Profile and roughness curves of 120-mesh sandpaper template.

Table S1 The optimization of PVA-PAA-Zr^{4+} hydrogel sheets

10 wt% PVA (g)	Glycerol/water (g)	AA (g)	ZrCl4 (mg)	TEMED (µL)	APS (mg)	Peelability	Stretchability
0	3/9	2	75	40	44	Hard	Inadequate
1	3/8	2	75	40	44	Hard	Inadequate
2	3/7	2	75	40	44	Hard	Adequate
3	3/6	2	75	40	44	Hard	Adequate
5	3/4	2	75	40	44	Fair	Adequate
7	3/2	2	75	40	44	Nearly good	Adequate
8	3/1	2	75	40	44	Good	Inadequate
7	3/2	2	100	40	44	Good	Adequate
7	3/2	2	150	40	44	Good	Inadequate

Table S2 Summary for performance of strain sensors based on conductive hydrogels containing carbon nanotubes

	Detection limit	GF (Strain range)	Conductivity	Response/recovery (time)	Frost resistance
**MCNT/OH/AAm/PVA [53]**	5%	2.52(0-200%)<br>6.39(200-560%)	5.6ms/cm	80/60 ms	-20℃
**CNT/PAAC/SA/P(AAM-co-HEMA) [54]**	---	1.1(0-100%)<br>2.6(100-150%)<br>3.1(150-200%)	2.1ms/cm	---	-20℃
**PAAm-oxCNTs [55]**	---	1.5(0-250%)<br>3.39(250-700%)	0.67ms/cm	300 ms/---	---
**CNTs/HAPAAm [56]**	10%	2(0-300%)<br>4.32(300-1000%)	---	180/200 ms	---
**PAM/Lap-IL/TA/c-MWCNTs [57]**	---	3.15(0-100%)<br>5.79(100-200%)<br>7.19(200-300%)<br>13.27(300-400%)<br>21.56(400-500%)	16.4ms/cm	200/200 ms	---
**MWCNTs Aerogel/PVA-PAA-Al^{3+} [26]**	0.1	29.34(1000%)	6.72 ms/cm	250/250 ms	-18℃
**LM@CNTs-PAA [58]**	---	2.49(0-300%)<br>15.4(300-500%)	---	649 ms/---	---
**PVA-CMCNFs [59]**	---	3.76(0-100%)	---	---	-20℃
**p(AAm/DMLB)/IL-LPs/c-MWCNTs [60]**	5%	12.71(1100%)	9 ms/cm	500 ms/---	-20℃
**SCNF-CNT/PAM [61]**	---	0.15(0-100%)<br>3.7(100-400%)	1.67 ms/cm	216/235 ms	---
**PAM/SA/CNTs/LM-Ag (microcrack) [62]**	10%	54.25 (0-300%)	---	149/167 ms	---
**micropatterned /MWCNTs/PVA-PAA-Zr^{4+} (This wok)**	0.1	76.1(0-30%)<br>203.5(30-100%)	278 mS/cm	250/250 ms	-20℃

Table S3 The maximum width of cracks in MWCNT layer on micropatterned composite hydrogel sheet under different strains

Strain (%)	0	5	15	30	40	50
Maximum width of cracks (μm)	0	8.47*3<br>12.7*4<br>16.95*4<br>21.19*6<br>29.66*7<br>38.14*2	16.95*2<br>33.9*5<br>80.51*3<br>131.36*2<br>164.55<br>206.19	80.51*4<br>101.69*4<br>122.88<br>152.54*4<br>241.56<br>372.88<br>483.05<br>516..96<br>597.46	144.07*3<br>152.54*2<br>279.66*4<br>394.07*2<br>453.39<br>662.22<br>788.14<br>906.78<br>983.05	207.63<br>250<br>330.51<br>364.41<br>491.53<br>576.27<br>669.49<br>741.53<br>1004.24
**Mean value (μm)**	**0**	**21.35**	**63.43**	**204.10**	**466.86**	**515.07**

Table S4 The optimization process of sandpaper template

Sandpaper sample	60-mesh	**120-mesh**	240-mesh
Peelability	Good	**Good**	Not so good
Micropattern of hydrogel	Intact	**Intact**	Not so intact
Stability of MWCNT layer/Micropattern maintenance	Nearly good/Intact	**Good/Intact**	Nearly good/Nearly lost micropattern

Table S5 Multi-layer packaging strategy of micropatterned composite hydrogel sheet

1st layer	Removable PVC film with Ra<1 μm	To protect MWCNT layer vis reducing abrasion
2nd layer	Hot-sealed EVOH-PE pouch	To prevent water loss of hydrogel
3rd layer	Silicone foam with cavity/XLPE bubble wrap	To buffer the compression and vibration
4th layer	PET tray, PCM packs and insulated corrugated box	To form a packaging box with controlled temperature

* PVC is polyvinyl chloride. EVOH-PE is polyethylene coated with ethylene-vinyl alcohol. XLPE is cross-linked PE. PCM is phase change material.
